# Supplementary material for: A Single α Helix Drives Extensive Remodeling of the Proteasome Lid and Completion of Regulatory Particle Assembly
Source: Cell. 2015 Oct 8;163(2):432–44. doi: 10.1016/j.cell.2015.09.022 (PMC4601081; doi:10.1016/j.cell.2015.09.022)
Supplement: Document S1. Supplemental Experimental Procedures and Table S2 [file mmc1.pdf]

Cell
Supplemental Information

A Single α Helix Drives Extensive Remodeling of the Proteasome Lid and Completion of Regulatory Particle Assembly

Robert J. Tomko, Jr., David W. Taylor, Zhuo A. Chen, Hong-Wei Wang, Juri Rappsilber, and Mark Hochstrasser

Supplemental Experimental Procedures

Purified LP2 and lid from yeast were prepared exactly as described previously (Tomko and Hochstrasser, 2011). Recombinant Rpn10 was purified on Ni-NTA resin (Qiagen) via an N-terminal 6His tag, followed by Sephacryl S-200 chromatography in Lid Buffer (50 mM HEPES•NaOH, pH 7.5, 100 mM NaCl, 100 mM KCl, 5% glycerol, 1 mM DTT). Recombinant base precursor bearing an N-terminal FLAG tag on Rpt1 and an N-terminal 6His tag on Rpt3 was expressed and purified exactly as described previously (Beckwith et al., 2013). Base precursor bearing an N-terminal MBP tag on Rpt1 and a C-terminal 6His tag on Rpn2 was purified exactly as for FLAG-tagged base precursor, except that the eluates from the Ni^{2+} affinity step were bound to amylose resin (NEB Corp.) in Base Buffer (50 mM HEPES•NaOH, pH 7.5, 100 mM NaCl, 100 mM KCl, 10 mM $MgCl_2$, 0.5 mM EDTA, 10% glycerol, 1 mM DTT), washed with Base Buffer, and eluted with Base Buffer containing 10 mM maltose instead of FLAG peptide.

Recombinant LP2, lid, and their derivatives were expressed in BL21-STAR (DE3) cells (Invitrogen) in Terrific Broth overnight at 16°C. For purification of complexes harboring an N-terminal MBP tag on Rpn6, cells in Lid Buffer supplemented with protease inhibitors were lysed using an M-110EH microfluidizer (Microfluidics Corp.). Lysates were cleared by centrifugation for 20 min at 30,000 x *g* at 4°C and bound to amylose resin. After washing extensively with Lid Buffer, proteins were eluted with 10 mM maltose in Lid Buffer, and separated by Superose 6 chromatography (GE Life Sciences) isocratically in Lid Buffer. The MBP tag was removed with human rhinovirus 3C protease overnight at 4°C, and the protease and cleaved tag were removed by Superose 6 chromatography as above. Polyhistidine-tagged proteins were purified as above, except that lysis and wash buffers for affinity purification contained 20 mM imidazole, Ni-NTA

resin was substituted for amylose resin, and proteins were eluted with Lid Buffer containing 500 mM imidazole. Complexes were then further purified by Superose 6 chromatography in Lid Buffer. All proteins were concentrated via centrifugal filtration using concentrators (Amicon) with an appropriate molecular weight cut-off for retention of the desired species, flash-frozen in liquid nitrogen, and stored at -80°C until use.

Single-particle pre-processing, alignment, and classification. All image pre-processing and two-dimensional classification was performed in Appion (Lander et al., 2009) as described previously (Lander et al., 2012). The contrast transfer function (CTF) of each micrograph was estimated, and particles were selected concurrently with data collection using ACE2 (Mallick et al., 2005) and a template-based particle picker (Roseman, 2004), respectively. Reference-free 2D class averages of lid and reference-free 2D class averages of LP2 were used to minimize reference bias. Micrograph phases were corrected using ACE2 (Mallick et al., 2005), and the negatively stained LP2/lid particles were extracted using a 288 × 288-pixel box size. The particle stacks were binned by a factor of 2 for processing, and particles were normalized to remove pixels whose values were above or below 4.5-σ of the mean pixel value using XMIPP (Sorzano et al., 2004). Particle stacks were binned again by a factor of 2 and subjected to five rounds of iterative multivariate statistical analysis (MSA) and multi-reference alignment (MRA) using the IMAGIC (van Heel et al.) software package to generate reference-fee class averages. The resulting set of class averages for each species was normalized using ‘proc2d’ in EMAN (Ludtke et al., 1999). The EMAN classification program ‘classesbymra’ was used to match each LP2 complex to the best-matching lid class average based on cross-correlation coefficients.

3D reconstruction and analysis. All three-dimensional reconstructions were performed using an iterative projection-matching refinement with libraries from the EMAN2 and SPARX software packages (Hohn et al.; Tang et al.). Refinement of the initial models began using an angular increment of 25°, progressing down to 4° for all reconstructions. The initial model used for each dataset was EMDB-1993 (Lander et al., 2012) low-pass filtered to 60-Å resolution. In an alternative approach for the lid, we used a low-pass filtered model of the other structure (LP2) after refinement as an initial model for the above-mentioned projection matching refinement. This led to an EM density with similar structural features (data not shown). Estimated resolutions were determined by calculating the Fourier shell correlation (FSC) between each of the back-projected volumes of two equally sized data sets. The final reconstructions of LP2 and lid showed structural features to ~16 Å and ~22 Å, respectively, based on the 0.5 Fourier shell correlation criterion (Fig. S3E). Reprojections of the final three-dimensional reconstruction of LP2 showed excellent agreement with the reference-free class averages (Fig. S3G) and displayed a large distribution of Euler angles, despite some preferential orientations of the particles on the carbon film (Fig. S3F).

The final reconstruction was segmented using Segger (Pintilie et al., 2010) in Chimera (Pettersen et al., 2004) based on inspection of the similarities between our LP2 reconstruction and the previously determined structure and segmentation of the lid (Lander et al., 2012). LP2 was superimposed onto the pseudo-atomic model of the 26S proteasome using a multi-step procedure. First, the previously determined lid structure was segmented and then docked into the lid subunits on the pseudo-atomic model of the 26S proteasome. Then, we docked our LP2 reconstruction into the lid density by optimizing the cross-correlation coefficient between the two EM densities. The N-terminal domain of Rpn6 from the pseudo-atomic model is not

accommodated by either of the EM structures, likely due to structural changes that occur upon Lid incorporation into the core particle as described previously (Lander et al., 2012).

Mass spectrometric analysis. We first excised cross-linked products of lid and LP2 from SDS-PAGE gels to obtain monomers, and not higher-order cross-linked forms, of each complex. The monomers of cross-linked lid and LP2 were then mixed after tryptic digestion. A precise 1:1 mixing was achieved based on a preliminary experiment in which we mixed small aliquots of each digested sample, conducted mass spectrometric measurements to determine actual molar ratios, and then used this information to normalize the protein amounts for mixing in the crosslink mapping analysis. Specifically, the digested samples were mixed in an approximately 1:1 ratio as estimated by the approximate protein concentrations used in the experiment. A short LC-MS analysis was conducted without any special instrument settings in order to detect linear modified peptides. Peptides were identified using Mascot version 2.4 and allowing for the cross-linker as a variable modification. Cross-linker modified peptides were then quantified by help of XiQ using the parameters described previously (Fischer et al., 2013). The median ratio for the modified peptides was used to correct the initial mixing ratio. Figure 2B illustrates the success of this procedure, as the quantified linkages center around log 2 = 0, i.e. 1:1 mixing ratio. To evaluate digestion efficiency, we used the number of uncut trypsin-cleavage sites in identified linear (non-cross-linked peptides) as an indicator of incomplete digestion. Few missed cleavages were found, suggesting similarly high and efficient digestion for both lid and LP2 samples.

LC-MS/MS analysis was conducted using a hybrid quadrupole-Orbitrap mass spectrometer (Q Exactive, Thermo Fisher Scientific). Peptides were separated on a reversed-phase analytical column (Chen et al., 2010). Mobile phase A consisted of water and 0.1% v/v

formic acid. Mobile phase B consisted of 80% v/v acetonitrile and 0.1% v/v formic acid. Peptides were loaded at a flow rate of 500 nl/min and eluted at 200 nl/min. The separation gradient consisted of a linear increase from 2% mobile phase B to 40% mobile phase B in 79 minutes and a subsequent linear increase to 95% B over 11 minutes. Eluted peptides were directly sprayed into the Q Exactive mass spectrometer. MS data were acquired in the data-dependent mode. For each acquisition cycle, the MS spectrum was recorded in the Orbitrap at 70,000 resolution. The ten most intense ions in the MS spectrum, with a precursor charge state 3+ or greater, were fragmented by Higher Energy Collision Induced Dissociation (HCD). The fragmentation spectra were thus recorded in the Orbitrap at 35,000 resolution. Dynamic exclusion was enabled, with single-repeat count and a 40 second exclusion duration.

Identification of cross-linked peptides. The raw mass spectrometric data files were processed into peak lists using MaxQuant version 1.2.2.5 (Cox and Mann, 2008) with default parameters, except that "Top MS/MS Peaks per 100 Da" was set to 20. The peak lists were searched against sequences of all lid subunits using Xi software (ERI, Edinburgh) for identification of cross-linked peptides. Search parameters were as follows: MS accuracy, 6 ppm; MS2 accuracy, 20 ppm; enzyme, trypsin; specificity, fully tryptic; allowed number of missed cleavages, four; cross-linker, BS^3/BS^3-d4; fixed modifications, carbamidomethylation on cysteine; variable modifications, oxidation on methionine. The linkage specificity for BS^3 was assumed to be for lysine, serine, threonine, tyrosine and protein N-termini. All identified cross-linked peptides were validated manually based on their MS2 spectra and MS signals (either as doublet signals or singlet signals with corresponding mass shift in label-swapped replica analysis Fig 2A).

Quantitation of cross-linking data. Identified cross-linked peptides were quantified based on their MS signals. Quantitative proteomics software tool Pinpoint was used to retrieve intensities of both light and heavy signals for each cross-linked peptides in an automated manner. An input library for cross-linked peptides was constructed according to "General Spectral Library Format for Pinpoint Comma Separated Values" (Thermo Fisher Scientific). The three most abundant isotope peaks were used for quantitation. The error tolerance for precursor m/z was set to 6ppm. Signals were only accepted within a window of retention time (defied in spectral library) ±10 minutes. Manual inspection was carried out to ensure the correct isolation of elution peaks. For each cross-linked peptide, elution peak areas of the light and the heavy signals were measured and signal fold changes were calculated as log2 (LP2/lid). Quantitation data was subsequently summarized at the level of unique residue pairs (cross-links). A cross-link was defined as a unique cross-link in either lid or LP2 only if all its supporting cross-linked peptides were observed as according singlet signals. Otherwise, cross-links were regarded as being observed in both particles. For a cross-link, the signal fold change was defined as the median of all its supporting cross-linked peptides. Only those cross-links that were consistently quantified in both paired replicas (with label-swap) were accepted for subsequent structural analysis. Singlet cross-links were further confirmed by a mass shift of 4 Da, resulting from the label-swap. For cross-links observed as a doublet, the average of fold ratios from replica analyses was reported. All quantified cross-links were listed in Supplementary Table S1. Cross-links that were significantly enriched in lid or LP2 were determined using "Significance A" test from the standard proteomics data analysis tool Perseus (version 1.4.1.2) (Cox and Mann, 2008) based on log2(LP2/lid) values. The following parameters were used for the test: "Side": both; "Use for truncation": P value; "Threshold value": 0.05.

Mapping and comparing cross-linking data with the pseudoatomic model of lid

To compare cross-linking data on the isolated lid with the structure of the lid in the context of the 26S proteasome, we mapped the identified and quantified lid cross-links onto the pseudoatomic structure of lid in the 26S proteasome structure (PDB 4CR2). Using PyMol (version 1.2b5) (DeLano, 2002), cross-links were displayed as lines between the C-α atoms of linked residues (cross-links were displayed only when both linked residues were present in the structure 4CR2). The distance of a cross-linked residue pair was measured between the C-α atoms. Measured distances of linked residue pairs in the pseudoatomic structure were compared to a theoretical cross-linking limit, which was calculated as side-chain length of cross-linked residues plus the spacer length of the cross-linker (11.4 Å for BS3). An additional 2 Å was added for each residue as an allowance for residue displacement from the pseudoatomic structure. The following side chain lengths were used for the calculation: 6.0 Å for lysine, 2.4 Å for threonine, 2.4 Å for serine and 6.5 Å for tyrosine. For example, for a lysine-lysine cross-link, this limit is 6+6+11.4+2+2=27.4 Å. While one might use a larger distance cut-off, we see most of our data in other studies where crystal structures were available to adhere to this upper limit. Also, the links that we observed in this study as over-length exceeded our upper limit by up to 99 Å and thus require the assumption of large conformational changes.

Supplemental Table S2 Related to Experimental Procedures. Plasmids used in this study.

Plasmid	Genotype
pRT205	pET28a-Rpn10
pRT585[a]	pET42b-Rpn3 : Sem1 : Rpn7
pRT586	pET42b-Rpn3 : Sem1 : Rpn7 : Rpn12
pRT945[a]	pCDF42b-6His-MBP-3Cx-Rpn6 : Rpn9 : Rpn11 : Rpn5 : Rpn8
pRT1010	pCDF42b-10His-EGFP-Rpn6 : Rpn9 : Rpn11 : Rpn5 : Rpn8
pRT1030	pCDF42b-6His-Rpn6 : Rpn9 : Rpn11 : Rpn5 : Rpn8
pRT1031	pCDF42b-6His-rpn6(173-434) : Rpn9 : Rpn11 : Rpn5 : Rpn8
pRT1032	pCDF42b-6His-rpn6(212-434) : Rpn9 : Rpn11 : Rpn5 : Rpn8
pRT1078	pET42b-Rpn3 : Sem1 : Rpn7 : rpn12(E271K)
pRT1096[b]	pCOLADuet-1-FLAG-Rpt1 : Rpt2 : 6His-Rpt3 : Rpt5 : Rpt6 : Rpt4
pRT1097[b]	pETDuet-1-Rpn1 : Rpn2 : Rpn13
pRT1098[b]	pACYCDuet-1-Nas6 : Nas2 : Hsm3 : Rpn14
pRT1122	pET42b-6His-Cys-rpn12(C23S,D265A)
pRT1202	pETDuet-1-Rpn1 : Rpn2-6His : Rpn13
pRT1260	pCOLADuet-1-MBP-3Cx-Rpt1 : Rpt2 : Rpt3 : Rpt5 : Rpt6 : Rpt4

[a] Described in Mol Cell. 2014;53:433-43.
[b] Described in Nat Struct Mol Biol. 2013; 20:1164-72.

Note: Unless otherwise indicated, all plasmids were produced in this study. 3Cx indicates a human rhinovirus 3C protease cut site.

Supplemental References

Chen, Z.A., Jawhari, A., Fischer, L., Buchen, C., Tahir, S., Kamenski, T., Rasmussen, M., Lariviere, L., Bukowski-Wills, J.C., Nilges, M., *et al.* (2010). Architecture of the RNA polymerase II-TFIIF complex revealed by cross-linking and mass spectrometry. EMBO J *29*, 717-726.

Cox, J., and Mann, M. (2008). MaxQuant enables high peptide identification rates, individualized p.p.b.-range mass accuracies and proteome-wide protein quantification. Nat Biotechnol *26*, 1367-1372.

DeLano, W.L. (2002). The PyMOL Molecular Graphics System.

Fischer, L., Chen, Z.A., and Rappsilber, J. (2013). Quantitative cross-linking/mass spectrometry using isotope-labelled cross-linkers. J Proteomics *88*, 120-128.

Hohn, M., Tang, G., Goodyear, G., Baldwin, P.R., Huang, Z., Penczek, P.A., Yang, C., Glaeser, R.M., Adams, P.D., and Ludtke, S.J. (2007). SPARX, a new environment for Cryo-EM image processing. J Struct Biol *157*, 47-55.

Lander, G.C., Stagg, S.M., Voss, N.R., Cheng, A., Fellmann, D., Pulokas, J., Yoshioka, C., Irving, C., Mulder, A., Lau, P.W., *et al.* (2009). Appion: an integrated, database-driven pipeline to facilitate EM image processing. J Struct Biol *166*, 95-102.

Ludtke, S.J., Baldwin, P.R., and Chiu, W. (1999). EMAN: semiautomated software for high-resolution single-particle reconstructions. J Struct Biol *128*, 82-97.

Mallick, S.P., Carragher, B., Potter, C.S., and Kriegman, D.J. (2005). ACE: automated CTF estimation. Ultramicroscopy *104*, 8-29.

Pettersen, E.F., Goddard, T.D., Huang, C.C., Couch, G.S., Greenblatt, D.M., Meng, E.C., and Ferrin, T.E. (2004). UCSF Chimera--a visualization system for exploratory research and analysis. J Comput Chem *25*, 1605-1612.

Pintilie, G.D., Zhang, J., Goddard, T.D., Chiu, W., and Gossard, D.C. (2010). Quantitative analysis of cryo-EM density map segmentation by watershed and scale-space filtering, and fitting of structures by alignment to regions. J Struct Biol *170*, 427-438.

Roseman, A.M. (2004). FindEM--a fast, efficient program for automatic selection of particles from electron micrographs. J Struct Biol *145*, 91-99.

Saeki, Y., Isono, E., and Toh, E.A. (2005). Preparation of ubiquitinated substrates by the PY motif-insertion method for monitoring 26S proteasome activity. Methods Enzymol *399*, 215-227.

Schlieker, C., Weihofen, W.A., Frijns, E., Kattenhorn, L.M., Gaudet, R., and Ploegh, H.L. (2007). Structure of a herpesvirus-encoded cysteine protease reveals a unique class of deubiquitinating enzymes. Mol Cell *25*, 677-687.

Sorzano, C.O., Marabini, R., Velázquez-Muriel, J., Bilbao-Castro, J.R., Scheres, S.H., Carazo, J.M., and Pascual-Montano, A. (2004). XMIPP: a new generation of an open-source image processing package for electron microscopy. J Struct Biol *148*, 194-204.

Tang, G., Peng, L., Baldwin, P.R., Mann, D.S., Jiang, W., Rees, I., and Ludtke, S.J. (2007). EMAN2: an extensible image processing suite for electron microscopy. J Struct Biol *157*, 38-46.

van Heel, M., Harauz, G., Orlova, E.V., Schmidt, R., and Schatz, M. (1996). A new generation of the IMAGIC image processing system. J Struct Biol *116*, 17-24.
