# Supplementary material for: A Single α Helix Drives Extensive Remodeling of the Proteasome Lid and Completion of Regulatory Particle Assembly
Source: Cell. 2015 Oct 8;163(2):432–44. doi: 10.1016/j.cell.2015.09.022 (PMC4601081; doi:10.1016/j.cell.2015.09.022)
Supplement: Document S2. Article plus Supplemental Information [file mmc4.pdf]

Cell

Article

A Single α Helix Drives Extensive Remodeling of the Proteasome Lid and Completion of Regulatory Particle Assembly

Graphical Abstract

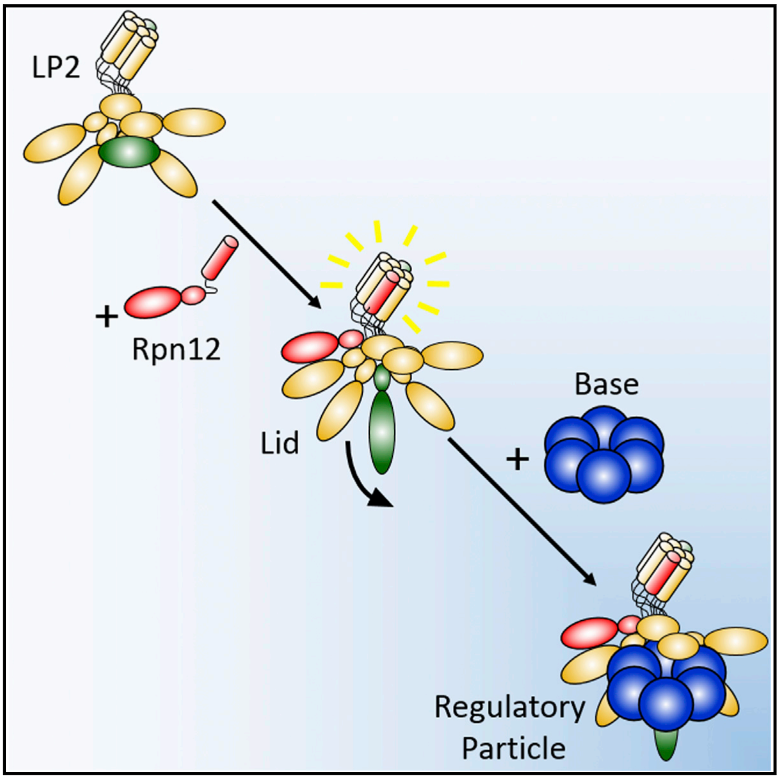

Highlights

- First in vitro reconstitution of RP assembly with completely recombinant components
- Electron microscopy and cross-linking reveal massive remodeling of a lid precursor
- Remodeling of the lid relieves steric clash with the RP base to promote RP assembly
- Lid remodeling can be triggered by a single C-terminal α helix in the Rpn12 subunit

Authors

Robert J. Tomko, Jr., David W. Taylor, Zhuo A. Chen, Hong-Wei Wang, Juri Rappsilber, Mark Hochstrasser

Correspondence

robert.tomko@med.fsu.edu (R.J.T.),
mark.hochstrasser@yale.edu (M.H.)

In Brief

A single alpha helix from the final subunit that incorporates into the proteasomal lid triggers a large-scale conformational switch that enables subsequent assembly of the lid and base, suggesting a general paradigm for hierarchical assembly of macromolecular complexes similar to that of virus particles.

CrossMark

Tomko et al., 2015, Cell *163*, 432–444
October 8, 2015 ©2015 The Authors
http://dx.doi.org/10.1016/j.cell.2015.09.022

CellPress

Cell

Article

A Single α Helix Drives Extensive Remodeling of the Proteasome Lid and Completion of Regulatory Particle Assembly

Robert J. Tomko, Jr.,[1,7,*] David W. Taylor,[2] Zhuo A. Chen,[3] Hong-Wei Wang,[4] Juri Rappsilber,[3,5,6] and Mark Hochstrasser[1,6,*]

[1]Department of Molecular Biophysics and Biochemistry, Yale University, New Haven, CT 06520-8114, USA
[2]California Institute for Quantitative Biosciences, University of California, Berkeley, Berkeley, CA 94720-3200, USA
[3]Wellcome Trust Centre for Cell Biology, University of Edinburgh, Michael Swann Building, King's Buildings, Max Born Crescent, Mayfield Road, Edinburgh EH9 3BF, Scotland
[4]Tsinghua-Peking Joint Center for Life Sciences, School of Life Sciences, Tsinghua University, Beijing 100084, PRC
[5]Department of Bioanalytics, Institute of Biotechnology, Technische Universität Berlin, 13355 Berlin, Germany
[6]Co-senior author
[7]Present address: Department of Biomedical Sciences, Florida State University College of Medicine, Tallahassee, FL 32306-4300, USA
*Correspondence: robert.tomko@med.fsu.edu (R.J.T.), mark.hochstrasser@yale.edu (M.H.)
http://dx.doi.org/10.1016/j.cell.2015.09.022
This is an open access article under the CC BY license (http://creativecommons.org/licenses/by/4.0/).

SUMMARY

Most short-lived eukaryotic proteins are degraded by the proteasome. A proteolytic core particle (CP) capped by regulatory particles (RPs) constitutes the 26S proteasome complex. RP biogenesis culminates with the joining of two large subcomplexes, the lid and base. In yeast and mammals, the lid appears to assemble completely before attaching to the base, but how this hierarchical assembly is enforced has remained unclear. Using biochemical reconstitutions, quantitative cross-linking/mass spectrometry, and electron microscopy, we resolve the mechanistic basis for the linkage between lid biogenesis and lid-base joining. Assimilation of the final lid subunit, Rpn12, triggers a large-scale conformational remodeling of the nascent lid that drives RP assembly, in part by relieving steric clash with the base. Surprisingly, this remodeling is triggered by a single Rpn12 α helix. Such assembly-coupled conformational switching is reminiscent of viral particle maturation and may represent a commonly used mechanism to enforce hierarchical assembly in multisubunit complexes.

INTRODUCTION

The eukaryotic proteome is constantly remodeled to control cell function. This remodeling depends heavily on protein degradation by the ubiquitin-proteasome system (UPS) (Finley, 2009; Ravid and Hochstrasser, 2008). The UPS consists of a cascade of enzymes that catalyze the transfer of the protein ubiquitin to protein substrates destined for degradation by the 26S proteasome. The proteasome is a 2.5 MDa ATP-dependent protease complex composed of a barrel-shaped proteolytic core particle capped on its open ends by 19S regulatory particles (Tomko and Hochstrasser, 2013). Under certain conditions, the regulatory particles (RP) can be divided into subcomplexes called the lid and base (Glickman et al., 1998). The lid consists of nine subunits, Rpn3, 5–9, 11, 12, and Sem1. The base contains a ring of six AAA+ family ATPases, Rpt1–6, and the non-ATPase subunits Rpn1, 2, 10, and 13. The lid removes the ubiquitin tag from substrates, whereas the base uses mechanical energy derived from ATP hydrolysis to unfold substrates and insert them into the core particle (CP) for destruction (Nyquist and Martin, 2014; Tomko and Hochstrasser, 2013).

The proteasome consists of at least 33 distinct subunits. Its assembly is a tightly coordinated process that relies both on dedicated extrinsic assembly chaperones and intrinsic features of the subunits themselves. Whereas both the CP and RP base depend heavily on assembly chaperones for efficient assembly, the lid can assemble independently of any additional eukaryotic factors (Fukunaga et al., 2010; Tomko and Hochstrasser, 2011, 2014). Lid biogenesis appears to follow a defined assembly sequence that culminates with addition of the Rpn12 subunit to a nearly complete lid intermediate consisting of Rpn3, 5–9, 11, and Sem1, called lid particle 2 (LP2) (Tomko and Hochstrasser, 2011). Recombinant lid forms efficiently in the absence of the base or CP (Lander et al., 2012; Tomko and Hochstrasser, 2014). Importantly, LP2 is unable to participate in further 26S proteasome assembly unless Rpn12 is present to complete lid formation (Tomko and Hochstrasser, 2011).

Incorporation of Rpn12 into LP2 licenses the resultant complex for assembly into full proteasomes, but the molecular mechanism underlying this critical function for Rpn12 has remained obscure (Tomko and Hochstrasser, 2011). In the mature 26S proteasome, Rpn12 occupies a peripheral position within the RP (Figures 1A and 1B) and contributes minimally to the interface between lid and base (Matyskiela et al., 2013; Unverdorben et al., 2014). Despite this, the LP2 intermediate has no detectable affinity for the base in the absence of the latter subunit (Tomko and Hochstrasser, 2011).

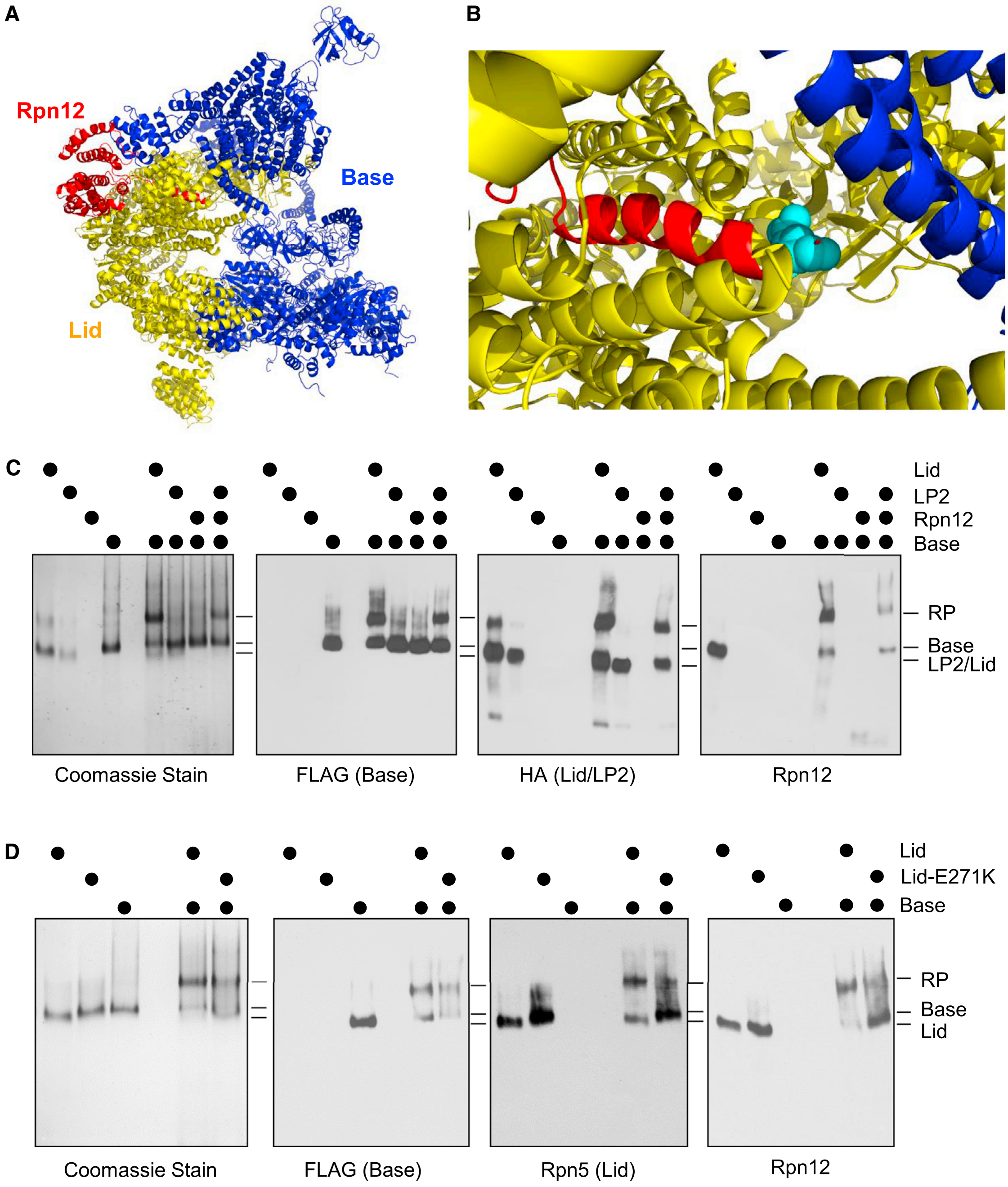

Figure 1. Reconstitution of the 19S Regulatory Particle Using Purified Components

(A) Pseudoatomic model of the proteasome regulatory particle (RP) (from PDB 4CR2) indicating the position of Rpn12 (red) with respect to (yellow) and base (blue) subunits. For clarity, the CP, as well as the RP subunits Rpn1 and Rpn10, is omitted.

(B) The Rpn12-Glu271 residue (shown in cyan) lies near the lid-base interface. CP, Rpn1, and Rpn10 have been omitted as in (A).

(C) RP assembly depends on Rpn12. The indicated recombinant components (1 μM) were incubated together in the presence of 1 mM ATP and 10 μM recombinant Rpn10 for 20 min at 30°C before separation by native PAGE. Gels were either stained with Coomassie brilliant blue or transferred to PVDF membranes followed by immunoblotting with antibodies to FLAG (on the Rpt1 base subunit), HA (on the Rpn7 LP2 subunit), or Rpn12.

(D) The rpn12-E271K mutation weakens lid-base interaction without interfering with lid formation. The indicated components (1 μM) were incubated with 1 mM ATP and 10 μM Rpn10 before analysis as in (C).

See also Figure S1.

Proteasomes have recently emerged as important targets for the treatment of certain cancers (Crawford and Irvine, 2013). All of the anti-proteasome drugs currently FDA approved or in clinical trials are inhibitors of the CP active sites (Dou and Zonder, 2014), but drug resistance is already a significant problem (Cheriyath et al., 2007; Richardson et al., 2003). Interference with proteasome assembly could provide a valuable alternative strategy for chemotherapy. As incorporation of Rpn12 is obligatory for subsequent RP assembly, interference with this final step of lid assembly would be an attractive target for such therapies. However, this will require more detailed knowledge of lid biogenesis and a better understanding of how Rpn12 regulates RP assembly. Thus, a central challenge in studying proteasome biogenesis, as well as that of many other multisubunit complexes, is to understand precisely how hierarchical assembly events are enforced in vivo.

Single-particle electron microscopy (EM) and mass spectrometry (MS) are powerful tools to investigate large macromolecular structures. Although these approaches have been used extensively with the fully assembled 26S proteasome (Lander et al., 2012; Sharon et al., 2006, 2007; Unverdorben et al., 2014; Witt et al., 2006), little information is available on the structures of proteasome assembly intermediates (Kock et al., 2015). Application of structural methods to assembly intermediates could provide substantial insight into the molecular mechanisms guiding proteasome biogenesis.

Quantitative cross-linking/MS (QCLMS) has recently emerged as a means to provide insights into dynamic aspects of protein structures (Fischer et al., 2013). An early application revealed structural changes within a multi-protein complex upon phosphorylation (Schmidt et al., 2013). Here, using a newly established workflow, we apply QCLMS to both the LP2 assembly intermediate and full lid; this has revealed multiple conformational changes, including the repositioning of the Rpn8-Rpn11 deubiquitinating enzyme (DUB) module. In addition, by comparing EM-derived molecular models of LP2, the free lid and the lid docked within the 26S proteasome, we show that incorporation of Rpn12 results in large-scale conformational remodeling of the nascent lid from a compact "closed" state in LP2, which is autoinhibited for base binding, to an open, more flexible state that can pair with the base. Remarkably, the highly conserved C-terminal α helix of Rpn12 is sufficient to promote conversion of LP2 to a lid-like conformation, enabling assembly of LP2 into the RP. These findings rationalize previous biochemical and genetic data indicating a critical role for Rpn12 in RP assembly, and provide novel insights into chaperone-independent proteasome assembly. Coupling small, local structural switches to large conformational changes is reminiscent of certain steps in virus assembly. Such locally induced conformational rearrangements are likely to be used in the hierarchical assembly of other multisubunit complexes, such as snRNPs, group II chaperonins, and ribosomes.

RESULTS

Reconstitution of Proteasome RP Assembly from Recombinant Constituents

Previously, we found that purified yeast LP2 assembled into full proteasomes when added to a yeast extract containing wild-type Rpn12, but not one with an Rpn12 truncation lacking C-terminal residues 212–274 (Figure S1) (Tomko and Hochstrasser, 2011). This could be due to a requirement for full-length Rpn12 or from an inhibitory effect of the truncated Rpn12 protein. Assembly might also require other factors in the extracts such as molecular chaperones. Thus, we sought a system of reduced complexity to analyze RP assembly. Toward this end, we established an in vitro RP assembly assay that consisted exclusively of bacterially expressed recombinant proteins.

Interaction of recombinant LP2 and a recombinant base precursor containing the Rpn14, Nas6, and Hsm3 assembly chaperones (hereafter called base) (Beckwith et al., 2013) was entirely dependent on Rpn12 (Figure 1C). Notably, Rpn12 could be supplied as part of the lid or added ectopically to the LP2 and base mixture with comparable results. In the pseudoatomic structure of the 26S proteasome determined by cryo-EM (PDB 4CR2), Rpn12 Glu271 lies near the interface between the lid and base (Figure 1B). As previously observed in yeast cells (Tomko and Hochstrasser, 2011), mutation of this highly conserved residue to lysine also impairs lid-base attachment in the in vitro system, but it does not compromise the stability of Rpn12 within the lid (Figure 1D). These findings support our previous observations that Rpn12 incorporation controls lid-base association in yeast and is regulated at least in part by the C-terminal helix of Rpn12.

QCLMS Reveals Local LP2-Lid Structural Changes

Rpn12 occupies a peripheral position within the RP and provides a very small fraction of the total interface between the lid and base complexes. This suggests that Rpn12 may instead promote lid-base joining primarily by inducing conformational changes in the assembling lid. To help determine how Rpn12 mediates lid-base joining, we first utilized QCLMS (Fischer et al., 2013) to identify protein cross-links that were unique or enriched in LP2 or lid, with the expectation that these differences reflect differences between the solution state conformations of these two particles. We purified each of the complexes from yeast (Figures S2A and S2B) and subjected them to cross-linking with non-deuterated or deuterated forms of the amine-reactive cross-linker BS^3, followed by protease digestion, 1:1 sample mixing, and LC-MS/MS analysis (Figure 2A). We used conditions identified in preliminary experiments that yielded only intra-complex cross-links (Figures S2C and S2D). The cross-linking experiments were done twice but with the non-deuterated and deuterated BS^3 swapped between LP2 and lid samples; this replicate analysis with label-swap controlled for any labeling bias and ensured accurate quantitation, especially for cross-links that are unique in either lid or LP2 (Figures S2E–S2G).

We identified and quantified 122 unique cross-links (Table S1). Among them, 81 were quantified consistently in label-swap replicas. Most of these 81 cross-links showed nearly equal prevalence in the lid and LP2 samples (i.e., were centered on $\log_2 = 0$; Figure 2B, i, and Figures S2E and S2F), which is consistent with 1:1 sample mixing (Figure 2B). However, one cross-link was unique to lid, and three were unique to LP2 (Figure 2B, ii). In addition, 16 of the 77 cross-links observed for both lid and LP2 were significantly enriched in either lid or LP2 ("Significance A" test, Perseus version 1.4.1.2 [Cox and Mann, 2008]; $p < 0.05$). Of these 16 cross-links, 15 changed more than 4-fold in at least

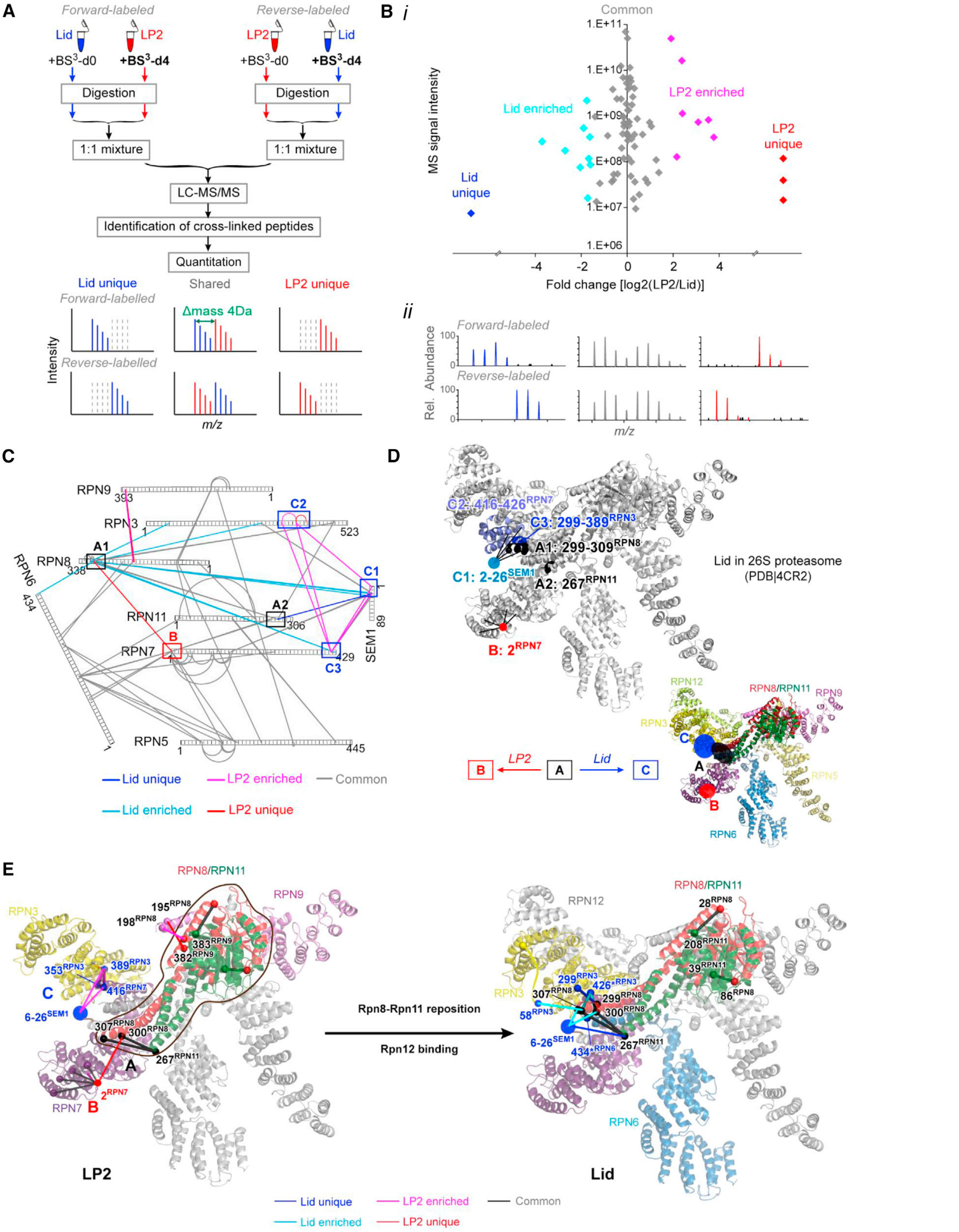

(legend on next page)

one of the replicated analyses (Figure S2F). Importantly, none of these cross-links directly involved Rpn12 or were in positions expected to be sterically occluded by Rpn12 based on the pseudoatomic structure of the 26S proteasome, strongly suggesting that these differences resulted from structural variance between the two particles.

Examination of the cross-links enriched in either LP2 or the lid suggested two major conformational differences between the two particles. The first was the relative positioning of the Rpn8-Rpn11 module (regions A1 [Rpn8 residues 299–309], A2 [Rpn11 residue 267], and B [Rpn7 residue 2], Figures 2C and 2D) within the complexes, and the second was the positioning of the Sem1 N terminus, the N-terminal α-helical domain of Rpn3, and the C-terminal α-helix of Rpn7 relative to one another (regions C1 [Sem1 residues 2–26], C2 [Rpn7 residues 416–426], and C3 [Rpn3 residues 299–389], Figure 2C). The latter three subunits yielded a web of cross-links that was selectively enriched in LP2, suggesting that these regions were more closely packed in LP2 (pink lines between regions C1, 2, and 3, Figure 2C). The enrichment of cross-links between the C-terminal helix of Rpn7 and residues in the N-terminal extension of Rpn3 (Figure S1) suggests that the helical bundle, or at least the C-helix of Rpn7, may be compacted against the body of Rpn3 and/or Rpn7 in LP2, and that it is repositioned upon incorporation of Rpn12. As Rpn12 forms an extensive interface with Rpn3 and appears to make little to no contact with Rpn7 in the 26S proteasome structure (Matyskiela et al., 2013; Unverdorben et al., 2014), it is likely that these changes are mediated through Rpn3.

Regarding the Rpn8-Rpn11 module, cross-links between Rpn8 and the C-terminal helix of Rpn9 (Figure S1) were enriched or unique to LP2 (Figure 2C), and an LP2-specific cross-link between Rpn8 and the extreme N terminus of Rpn7 was observed (red line between A1 and B, Figure 2C). In contrast, we observed lid-enriched cross-linking between the C terminus of Rpn8 and the C-terminal helix of Rpn7, the N-terminal region of Rpn3, and the N-terminal region of Sem1 (regions C1 and C3, light blue lines, Figure 2C). Further, a cross-link between the C-terminal helix of Rpn11 and the N-terminal region of Sem1 was detected only in the lid (regions A2 and C1, dark blue line, Figure 2C). A series of common cross-links between Rpn8 and Rpn11 suggests that the module itself is unlikely to vary substantially in structure between LP2 and lid.

Taken together, the enrichment of cross-linking in different regions of the lid and the loss or depletion of cross-linking to Rpn9 suggest that the Rpn8-Rpn11 module undergoes substantial movement upon incorporation of Rpn12 (Figure 2D). We suggest that a rigid-body rotation of the Rpn8-Rpn11 dimer (modeled in Figure 2E) may occur upon incorporation of Rpn12 that would account for the observed changes in cross-linking.

The N Terminus of Rpn5 Is Highly Flexible in the Undocked Lid

Currently, the only structural information on the isolated lid is from negative-stain EM (Lander et al., 2012). However, this structure is not at sufficiently high resolution to aid in visualizing our cross-linking data. To provide insight into the solution conformation of the free lid, we mapped our cross-link network for the isolated lid onto the lid within the cryo-EM structure of the 26S proteasome (Unverdorben et al., 2014). 61 cross-links could be mapped onto this structure (Figure 3, Data S1, and Table S1). Surprisingly, 26 of the 61 cross-links spanned distances greater than the calculated maximum distance between α-carbons in BS^3-cross-linked residues (based on spacer length of BS^3 and side-chain lengths of cross-linked residues; Supplemental Experimental Procedures) (Figure 3B), suggesting that the free lid is much more flexible than the docked lid. Two clusters of over-length inter-subunit cross-links were apparent. The first consisted of five cross-links between Rpn9 and either Rpn8 or Rpn11, indicating that the Rpn8/Rpn11 dimer is positioned closer to Rpn9 in the free lid than in the mature proteasome. Importantly, together with the results of our LP2-lid comparison, this further supports a model in which Rpn8/Rpn11 undergoes substantial movements during RP assembly.

The second cluster involved 19 cross-links between the N-terminal extension of Rpn5 and either itself or other lid subunits, with several of these cross-linked residues being over 100 Å apart in the 26S proteasome. Further, cross-links involving Rpn5-K108 were observed with residues from four subunits (Rpn3, Rpn6, Rpn8, and Rpn11) that are, in some cases, very far from one another in the proteasome-docked lid, suggesting substantial mobility of this domain of Rpn5 in the free lid. This was further supported by the observation that over-length intra-subunit cross-links were observed only within Rpn5 and not within any other subunit. Taken together, these data strongly suggest that the lid Rpn5 N-terminal domain is highly flexible

Figure 2. Quantitative Cross-linking/Mass Spectrometry Reveals Local Differences in LP2 and Lid Structures

(A) Overview of quantitative cross-linking/MS analysis of LP2 and lid complexes.

(B) *i*: fold changes [$\log_2$(LP2/lid)] of 81 quantified cross-links (consistently quantified in both forward- and reverse-labeled experiments) were plotted against their signal intensities. These quantified cross-links were divided into five subgroups and colored accordingly—three are unique to the LP2 sample (red), one is unique to the lid sample (blue); among 77 cross-links observed in both samples, 61 common cross-links showed no significant difference in yields in the LP2 and the lid samples (gray), while seven cross-links are significantly upregulated in the LP2 sample ($p < 0.05$, magenta) and nine in the lid sample ($p < 0.05$, cyan). *ii*: observed MS signals of supporting cross-linked peptides of "lid unique" cross-link 267^{RPN11}-20^{SEM1} (left), "common" cross-link 404^{RPN5}-150^{RPN11} (middle), and "LP2 unique" cross-link 2^{RPN7}-300^{RPN8} (right).

(C) Cross-linking network of lid/LP2 subunits. Proteins are shown as bars, and cross-links are shown as lines that are colored according to their quantified subgroups (described in B). LP2-unique, LP2-enriched, lid-unique, and lid-enriched cross-links, which suggest conformational differences between LP2 and lid, mainly involve three regions marked as A, B and C.

(D) Relative cross-link enrichments suggest a repositioning of the Rpn8-Rpn11 heterodimer within the lid structure (derived from PDB 4CR2) upon Rpn12 incorporation that may be important for subsequent RP assembly steps. Regions A, B, and C are highlighted accordingly. Regions C1 and B that are not present in 4CR2 are located based on their cross-linking partners in the model.

(E) A rigid-body rotation of the Rpn8-Rpn11 upon Rpn12 incorporation may account for the observed change in the Rpn8-Rpn11 cross-link network.

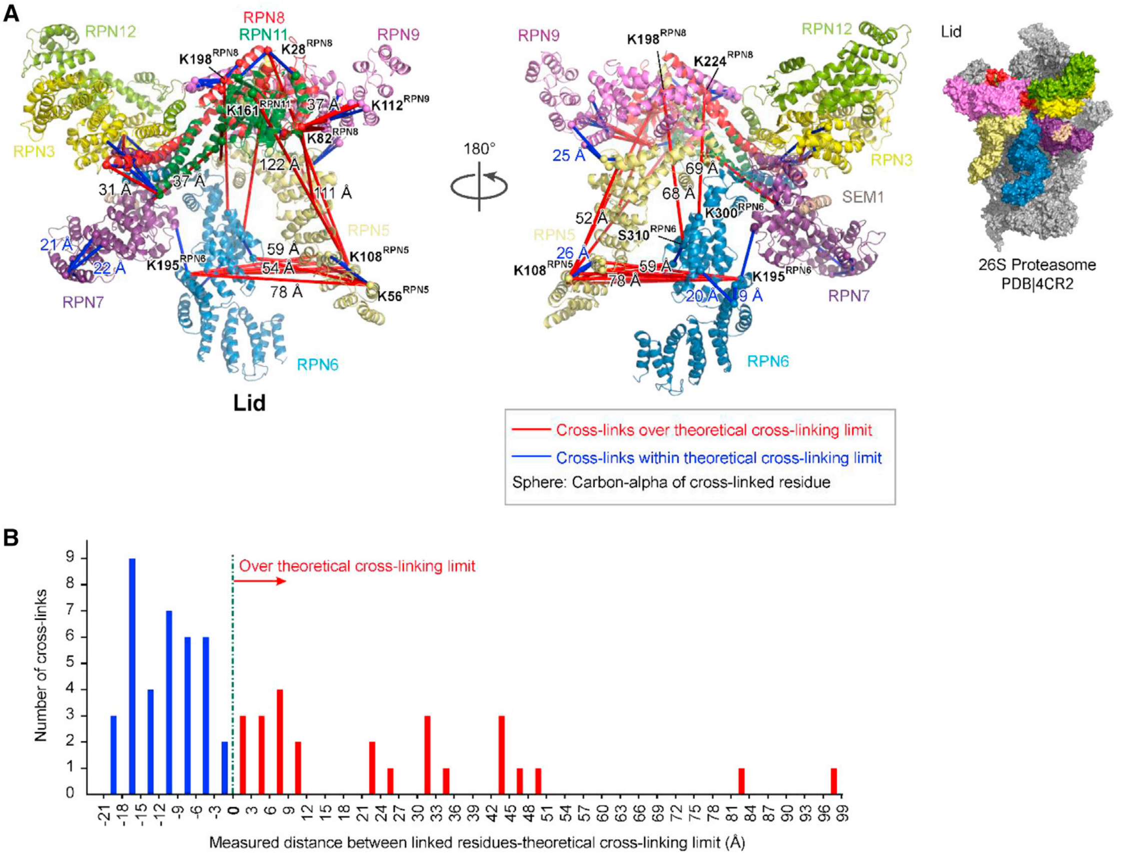

Figure 3. Mapping Lid Cross-links onto the Lid in the 26S Pseudoatomic Model Reveals Subunit Flexibility in the Undocked Lid

(A) Lid cross-links (61) detected in our QCLMS analysis are depicted on the 26S proteasome pseudoatomic structure (PDB 4CR2). Cross-links are displayed as straight lines connecting cross-linked residues, and distances between them are measured between Cα atoms. Cross-links are displayed only when both cross-linked residues are present in the proteasome pseudoatomic structure. Cross-links exceeding the predicted limit are shown in red, whereas those within the limit are shown in blue.

(B) The differences in length between the 61 cross-links mapped onto PDB 4CR2 in (A) and the theoretical cross-linking limit (e.g., 27 Å for lysine-lysine cross-links, Supplemental Experimental Procedures) are shown. Distances are between the Cα atoms of cross-linked residues.

See also Figure S2.

and free to undergo substantial movement in solution (Lander et al., 2012). Incorporation of the lid into the 26S proteasome likely stabilizes Rpn5 in the extended conformation via interaction of its N terminus with the base and CP and is accompanied by a repositioning of the Rpn8/Rpn11 module away from Rpn9.

EM Structure of LP2 Reveals a Compact Intermediate

We next investigated the architecture of LP2 by negative-stain EM and single-particle analysis for comparison to the negative stain EM structure of the isolated lid (EMD-1993) (Lander et al., 2012). Raw micrographs of LP2 showed monodisperse particles with dimensions ≈200 Å × 250 Å (Figure S3A). Reference-free two-dimensional (2D) alignment and classification yielded class averages with strong similarities to the previously determined structure of the isolated lid (Figure 4A). As expected, LP2 lacked density for Rpn12, but surprisingly, also lacked the prominent N-terminal extension of Rpn6 that was previously observed in the isolated lid (Lander et al., 2012). We saw this absence of density for the N-terminal portion of Rpn6 both for LP2 purified from yeast, as well as recombinant LP2 produced in *E. coli* (Figures S3G and S3H).

To address whether the observed LP2-lid differences were a result of sample preparation differences, we repeated the EM analysis with the full lid. 2D class averages of lid purified from yeast showed a density for the N-terminal extension of Rpn6 (Figure 4A). Additionally, a three-dimensional (3D) reconstruction of lid obtained with our complexes was nearly identical to that determined by Lander et al. (2012) (Figures 4B, S3C, and S3D), indicating that sample preparation was not responsible for the structural differences between LP2 and lid. Using iterative back-projection refinement of LP2 particles with the structure of lid low-pass filtered to 60 Å as a starting model, we obtained a 3D reconstruction of LP2 at 16 Å resolution, showing the clear domain architecture of the complex (Figures 4B and S3E–S3G).

The quality of our LP2 structure allowed facile automated segmentation based on visual inspection and comparison to the previously determined lid density (Figure 4B). In agreement with our 2D analysis and protein cross-linking, the overall subunit

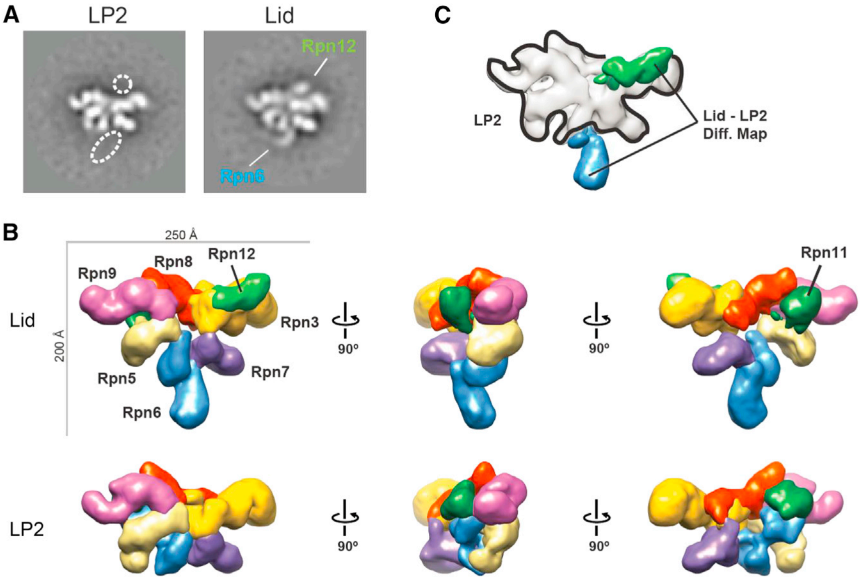

Figure 4. Electron Microscopy of LP2 and Lid Purified from Yeast Reveals Closed and Open Configurations, Respectively

(A) The N-terminal domain of Rpn6 adopts an extended conformation in lid, but not in LP2. Representative 2D class averages of LP2 or lid indicating the positions of Rpn12 and the Rpn6 N terminus are shown. The width of the boxes is ~430 Å.

(B) 3D reconstructions of the lid (EMD-1993) and LP2 (this study) are shown.

(C) Difference-density map of lid and LP2 indicates that the two major regions of variant density are those for Rpn12 and the N-terminal domain of Rpn6.

See also Figure S3.

architectures of LP2 and lid were very similar. In LP2, the Rpn6 N-terminal helical domain (Rpn6-N) is folded into a cavity formed by Rpn3, Rpn7, Rpn8, and Rpn11. Using 3D difference mapping between our LP2 structure and the lid structure from Lander et al. (2012), we unambiguously assigned the most prominent changes in density in the lid to Rpn12 and Rpn6-N (Figure 4C). This observation prompted us to reexamine our QCLMS data. Although the Rpn6-N conformation seen by EM in LP2 was not overtly reflected in LP2-specific cross-linking of this domain (Figure 3), an LP2-unique cross-link was in fact observed between Rpn6-Lys195 and Rpn11-Lys12 (Table S1). This particular amino acid of Rpn11 is not modeled in the cryo-EM structure (PDB: 4CR2), but extrapolation from the most proximal modeled Rpn11 residue (Thr23) indicates that the cross-link between Rpn6 and Rpn11 must be well over the maximal predicted cutoff. In further support of the compact conformation of LP2 observed by EM, two overlength cross-links were observed between Rpn6-N and Rpn8 (Rpn6-Lys300 to Rpn8-Thr224 and Rpn6-Ser310 to Rpn8-Lys198) for both the lid and LP2 (Figure 3A and Table S1). Formation of these cross-links in the lid, as well as LP2, suggests that the lid may dynamically sample more compact conformations.

LP2 Is Sterically Incompatible with Positioning in the Mature 26S Proteasome

In the 26S proteasome, the Rpn6 N-terminal helical domain is extended and makes contact with subunits of the RP base and CP (Lander et al., 2012; Pathare et al., 2012). Since this domain is folded into the core of LP2, it raised the possibility that adoption of this conformation produced a steric conflict with the base that would prevent incorporation of LP2 into the assembling RP prior to Rpn12 assimilation. By docking the previously determined EM structure of the isolated lid onto the lid subunits of the pseudoatomic model of the 26S and then overlaying our LP2 structure onto this structure, we were able to examine whether LP2 could incorporate into the proteasome without steric conflict (Figure 5A). In the EM structure of the 26S proteasome, Rpn3 and Rpn9 curve inward toward the center of the RP and make direct contacts with Rpn2 and Rpn10, respectively. In the LP2 model, Rpn3 and Rpn9 are not curved inward, which could contribute to the apparent poor affinity of LP2 for the base. Further, in LP2, Rpn6-N cannot make the contacts with the core particle and base ATPase ring that are observed for this domain in the mature 26S proteasome. Rather, the model displayed clear steric clashes between the LP2 Rpn6-N conformation and segments within the base ATPase subunits Rpt3, Rpt4, and Rpt6 (Figure 5A, inset). This strongly implies that the closed conformation observed for LP2 is an autoinhibited conformation that prevents lid-base association until Rpn12 incorporation. This model would rationalize the behavior of LP2 in vitro (Figure 1) and in yeast (Tomko and Hochstrasser, 2011).

Addition of Purified Rpn12 to LP2 Is Sufficient to Drive Lid Maturation

If Rpn12 relieves an autoinhibited conformation of LP2 to promote lid-base joining, then adding purified Rpn12 to LP2 should promote structural changes in LP2 that result in a lid-like structure. We treated purified recombinant LP2 with an equimolar concentration of recombinant Rpn12 and examined the structure of the resultant complex by EM. Consistent with a role for Rpn12 in maturation of the assembling lid, 2D class averages indicated that Rpn12 addition caused extension of Rpn6-N to a conformation similar to that observed in the purified full lid (Figure 5B). Thus, incorporation of Rpn12 drives a large-scale structural rearrangement of the assembling lid that relieves the autoinhibitory conformation of the Rpn6 N terminus.

Using two complementary approaches, we next addressed whether relieving the steric conflict between the N-terminal domain of Rpn6 and the base was sufficient to drive RP assembly in the absence of Rpn12. In the first, we appended the bulky GFP protein to the N terminus of Rpn6, which should physically clash with surrounding subunits in the closed conformation and potentially induce the extended conformation for Rpn6-N. In the

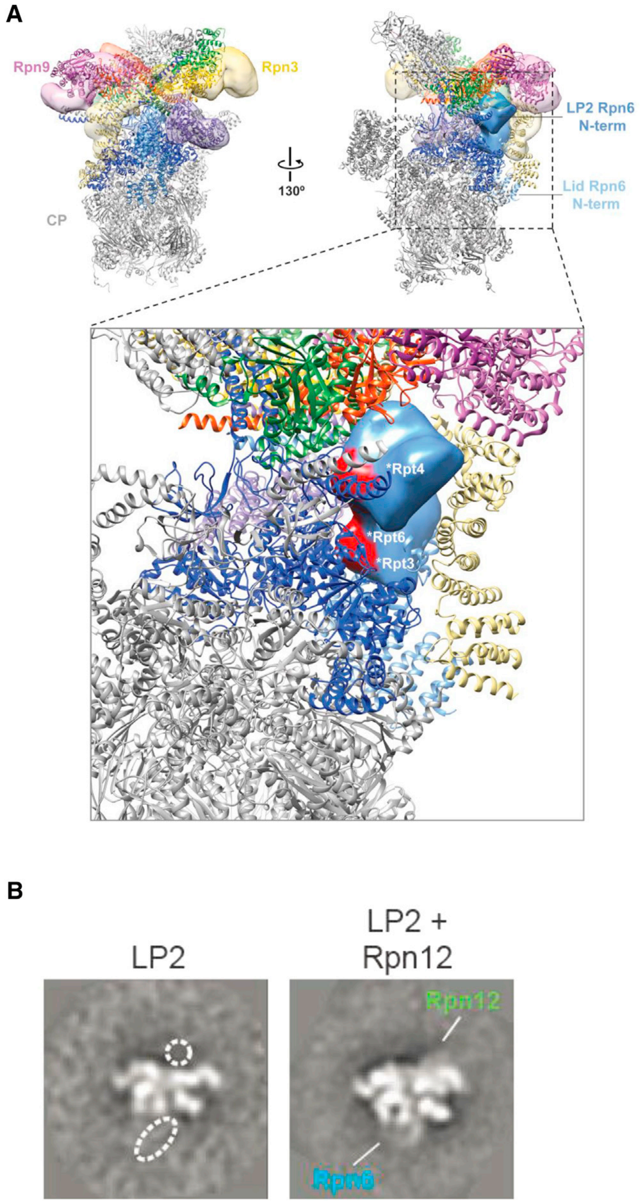

Figure 5. Rpn12 Addition Relieves an Autoinhibitory Conformation of the Rpn6 N Terminus

(A) The LP2 3D reconstruction (transparent surface) was modeled onto the pseudoatomic model of the 26S proteasome (PDB 4CR2) using the winged-helix horseshoe as a guide point, revealing steric clash (red surface) between the N-terminal domain of Rpn6 in LP2 (medium blue surface) and Rpt3, 4, and 6 (dark blue ribbons) of the base (inset). The corresponding position of the N-terminal domain of Rpn6 in the lid is also indicated (light blue ribbons). Rpn3 (yellow) and Rpn9 (magenta) are highlighted to show differences between LP2 and the docked lid.

(B) Addition of recombinant Rpn12 to purified recombinant LP2 triggers extension of the Rpn6 N terminus. The class averages are labeled as in Figure 4A. The width of the boxes is ~430 Å.

second approach, we engineered N-terminally truncated forms of Rpn6 that were anticipated to lack the putative autoinhibitory domain (Figure 6A). Both the GFP-Rpn6 fusion and N-terminal Rpn6 truncation proteins assembled efficiently into LP2 when coexpressed with the other LP2 subunits in *E. coli* (Figures S4A and S4B), suggesting that Rpn6-N is dispensable for LP2 assembly. As predicted, appending GFP to the N terminus of Rpn6 resulted in a conformation of Rpn6-N within LP2 that appeared similar to its extended conformation in the full lid (Figures 6B and S4C). Importantly, each of the mutant forms of LP2 assembled into the RP when recombinant Rpn12 was provided (Figure S4D), indicating that they were functional for RP assembly. In contrast, neither GFP-LP2 (Figure 6C) nor LP2 harboring truncations of Rpn6 (Figure 6D) assembled into an RP-like structure in the absence of Rpn12. Therefore, repositioning or removal of Rpn6-N is insufficient to promote LP2-base joining. This indicates that Rpn12 binding to LP2 triggers additional changes in the lid precursor that are necessary for RP formation.

Rpn12 C-Terminal Helix Engagement of the Lid Helical Bundle Licenses RP Assembly

The C terminus of Rpn12 forms an α helix that inserts into a pronounced groove within a helical bundle formed by C-terminal helices from all the other large lid subunits (Estrin et al., 2013). This helix of Rpn12 is important both for the completion of lid assembly and engagement of the lid with the base (Tomko and Hochstrasser, 2011). The complex interface between the helical bundle and the Rpn12 helix would in principle allow Rpn12 to sense and transmit signals through multiple lid subunits. We therefore asked whether provision of the Rpn12 C-terminal helix alone was sufficient to drive lid-base association.

A fluorescently labeled peptide composed of Rpn12 residues 254–272 (N12pep) bound to purified LP2 in vitro and could be competed off with excess recombinant Rpn12, indicating that the peptide bound LP2 in a manner similar to the full-length protein (Figure 7A). Indeed, addition of N12pep to purified LP2 triggered conformational extension of the Rpn6 N-terminal domain (Figure 7B), as had been observed with full-length recombinant Rpn12 (Figure 5B). Most importantly, N12pep promoted LP2-base joining when LP2 and N12pep were supplied in molar excess to the base, as revealed by a migration shift of the base on native gel blots (Figure 7C). Assembly into the RP was not as efficient as it was with recombinant Rpn12, suggesting that other parts of Rpn12 promote assembly, possibly by stabilizing the Rpn12 C-terminal helix. RP formation was specific to N12pep, as identical concentrations of two unrelated peptides had no effect on LP2-base joining (Figure 7D). In summary, our data indicate that engagement of the nascent lid helical bundle by the Rpn12 C-terminal helix is the key event triggering conformational remodeling of the assembling lid and subsequent lid-base joining.

DISCUSSION

Hierarchical assembly has emerged as a theme in the regulation of proteasome biogenesis (Tomko and Hochstrasser, 2013). In yeast and mammals, most evidence suggests that the proteasome lid assembles completely before making stable contact with the base complex or its subunits (Fukunaga et al., 2010; Thompson et al., 2009; Tomko and Hochstrasser,

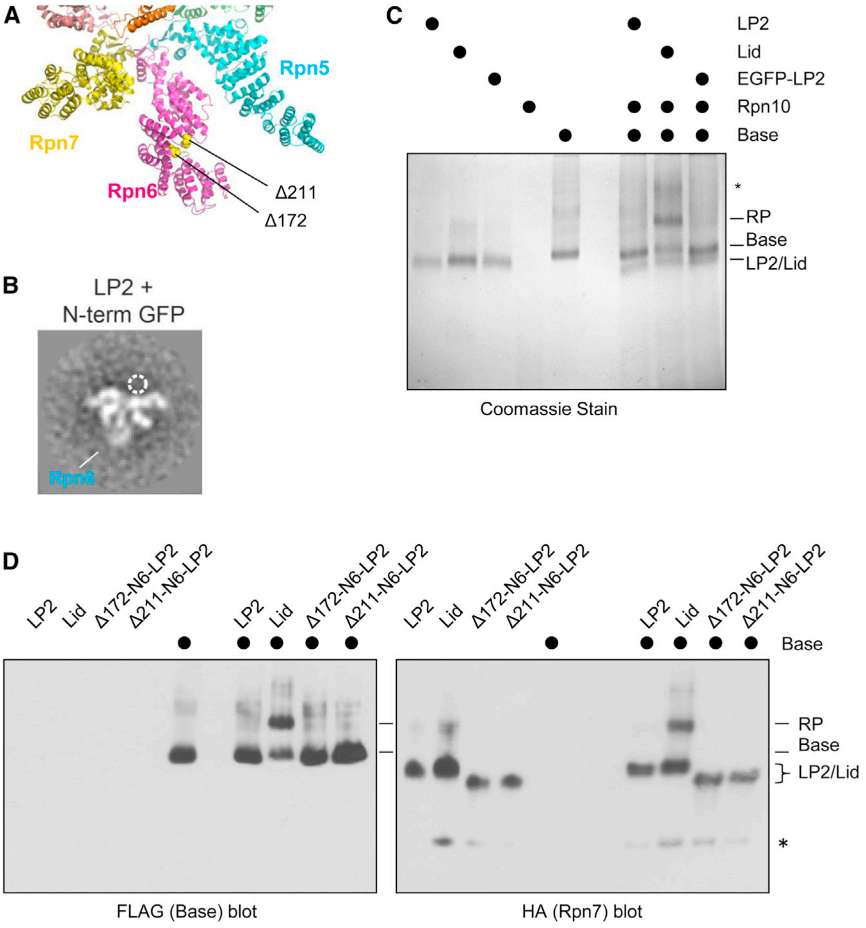

Figure 6. Relief of Steric Conflict Imposed by the Rpn6 N Terminus Is Not Sufficient to Drive Binding of LP2 to the Base

(A) The positions of truncations to Rpn6 are shown on the pseudoatomic structure of the lid from PDB 4CR2. The base and core particle subunits are omitted for clarity. The positions of Rpn6 amino acid 173 and Rpn6 amino acid 212 (the first endogenous residues present in the Δ172 and Δ211-rpn6 truncation mutants, respectively) are shown as yellow spheres.

(B) Fusion of GFP to the N terminus of Rpn6 within LP2 causes the N terminus to assume a conformation similar to that observed for the lid. The width of the box is ~430 Å.

(C) Addition of LP2 containing a GFP-Rpn6 fusion to the base precursor is not sufficient to trigger LP2-base association. Untagged LP2, LP2 with N-terminally GFP-tagged Rpn6 (GFP-LP2), or lid (1 μM each) were added to 1 μM base and 10 μM Rpn10 before analysis by native PAGE. Asterisk, poorly resolved or potentially aggregated protein(s).

(D) Addition of LP2 containing truncated forms of Rpn6 is not sufficient to trigger LP2-base association. Untagged LP2, LP2, lid, or the indicated forms of Rpn6 truncation mutant LP2 (1 μM each) were added to 1 μM base and 10 μM Rpn10 before analysis by native PAGE and immunoblotting. Asterisk, cross-reactive band.

See also Figure S4.

2011). The mechanism underlying this observation had been unknown. Our combined QCLMS, EM, and biochemical reconstitution studies now show that both large-scale conformational rearrangements and local repositioning of lid subunits are triggered by Rpn12 binding. These rearrangements underlie the coupling between completion of lid assembly and licensing of the lid for attachment to the base. Our findings rationalize previous genetic data implicating the Rpn12 C-terminal helix in RP formation and reveal how Rpn12 can govern lid-base joining despite making minimal contacts with the base in the mature proteasome.

Local and Global Conformational Changes during Lid Assembly

Mapping the quantified cross-links onto the 26S proteasome pseudoatomic structure reveals that the Rpn8/Rpn11 module is repositioned as assembly progresses from LP2 to lid and finally to the full proteasome (Figures 2D and 3). Recently, it has been shown that the isolated recombinant Rpn8/Rpn11 heterodimer has significant deubiquitinating (DUB) activity (Worden et al., 2014). Intriguingly, we did not detect DUB activity in LP2 (Figure S4E and data not shown) or in the full lid (data not shown). It is unclear whether the separate Rpn8-Rpn11 module occurs physiologically either as a lid assembly intermediate or a lid subcomplex with extra-proteasomal functions (Hofmann et al., 2009; Rinaldi et al., 2004, 2008), but we speculate that Rpn11 DUB may be held inactive in the context of LP2 and lid.

The repositioning suggested by our QCLMS data may be required for Rpn11 activation during incorporation into the proteasome holoenzyme, perhaps by relief of inhibitory contacts with other lid subunits. It is unlikely that Rpn6 would serve this autoinhibitory role given that Rpn6-N is extended away from the Rpn8/Rpn11 module in the EM structure of the lid. However, the high flexibility of Rpn5 (Figure 3) makes it an attractive candidate for this putative function. Notably, the Rpn5 paralog of the lid-related COP9/signalosome (CSN), CSN4, engages and inhibits the activity of the CSN6-CSN5 module (paralogous to Rpn8/Rpn11) in the fully assembled CSN (Lingaraju et al., 2014). This inhibition is released upon substrate engagement to stimulate the deneddylase activity of CSN5.

The lid is generally thought to interact stably with the base (and perhaps the CP), and Rpn11 DUB activity is regulated in part through ATP binding and/or hydrolysis by the base (Liu et al., 2006; Matyskiela et al., 2013; Verma et al., 2002). However, Rpn8 and Rpn11 make very little contact with base subunits in the proteasome holoenzyme. Our observation that Rpn12 binding to the lid at a remote location can reposition the Rpn8-Rpn11 module suggests the possibility that ATP binding or hydrolysis by the base might also allosterically regulate this module through movements of the lid rather than direct contacts with the ATPase heterohexamer itself.

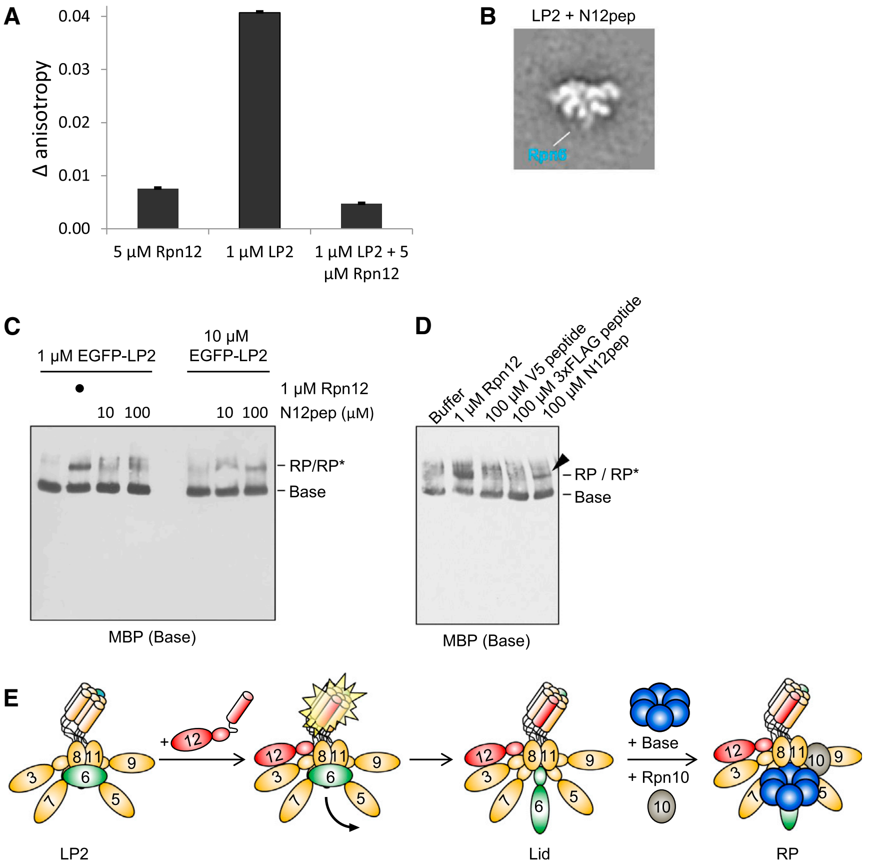

Figure 7. Engagement of the Lid Helical Bundle by the Rpn12 C-Terminal Helix Licenses the Lid for RP Assembly

(A) A fluorescently labeled peptide corresponding to amino acids 254–272 from yeast Rpn12 (N12pep) binds to LP2 similarly to full-length Rpn12. The average difference in fluorescence anisotropies between the free fluorescein-labeled N12pep and N12pep after addition of the indicated proteins are shown. Error bars, SEM.

(B) Addition of N12pep triggers extension of the Rpn6 N terminus from the body of LP2. N12pep (10 μM) was added to purified, untagged LP2, followed by analysis by negative-stain EM. The width of the box is ~430 Å.

(C) N12pep triggers association of LP2 and base in the absence of Rpn12. EGFP-LP2 (1 or 10 μM) was incubated with 1 μM base, 10 μM Rpn10, and the indicated concentration of N12pep before native PAGE and immunoblotting. RP*, an RP-like species devoid of Rpn12 but reactive with both lid (not shown) and base antibodies.

(D) N12pep, but not V5 or 3xFLAG control peptides, trigger LP2-BaseP association. LP2 harboring an N-terminal EGFP tag on Rpn6, base harboring an N-terminal MBP tag on Rpt1, and Rpn10 were incubated as in (C) with 100 μM of the indicated peptides, followed by analysis as in (C). An arrowhead marks RP*.

(E) Model for Rpn12-dependent licensing of the lid for lid-base joining. LP2 assumes a closed conformation with the N-terminal domain of Rpn6 folded inward, causing steric clash with the base. During incorporation of Rpn12 into LP2, engagement of the C-terminal helical bundle triggers extension of the Rpn6 N terminus, as well as additional, as-yet-unknown events (not depicted) that promote a conformation of the lid that is competent for binding the base.

The Rpn12 C-Terminal Helix as an Allosteric Trigger

Our EM analyses and reconstitution experiments demonstrate that the Rpn12 C-terminal helix initiates a global remodeling of the lid structure from an autoinhibited conformation to one that can join to the base. Importantly, this single helix is sufficient to promote both the remodeling of LP2, as well as interaction between LP2 and base. Although the peptide was not as potent as full-length Rpn12 in the base-joining assay, this could simply reflect a lower affinity of the isolated peptide for LP2 compared to Rpn12. Other elements of Rpn12 may enhance binding of the C-terminal helix to LP2 via Rpn12 interactions with Rpn3. Additional contacts between Rpn12 and LP2 (and base) may also be necessary to promote an optimal conformation of the lid for binding to the base.

We found that Rpn6-N undergoes a striking movement from a position pressed against other subunits in LP2 to an extended conformation in the lid. Computational docking of the LP2 model onto the 26S proteasome indicates that steric clash would occur between the base ATPase ring and Rpn6-N. As neither displacement nor removal of the putative autoinhibitory domain alone was sufficient to trigger stable interaction between LP2 and the base, additional changes must also be needed. Other large steric clashes were not readily apparent in our modeling of LP2 onto the 26S proteasome, but this could simply reflect differences in the conformation of the assembly chaperone-bound base precursor used in our study and the chaperone-free base in the context of the 26S holoenzyme observed in previous EM studies. No structural information currently exists on the chaperone-bound base precursor. The combination of QCLMS and EM approaches used here could be valuable in exploring the structure of the base precursor relative to the structure of the base in the context of the mature proteasome; such differences might also be important for regulating lid-base joining.

In this study, QCLMS was applied for the first time to study conformational changes involved in macromolecular assembly. Compared to a previous use of isotope-labeled cross-linkers for quantification (Schmidt et al., 2013), our workflow advanced a number of critical aspects: replicate analysis with label swap ensured accurate quantitation; summarizing quantitation into residue pairs reduced the impact of unrelated influences that would be felt at the peptide level (such as additional modifications or digestion efficiency); and use of well-established quantitative proteomics software minimized errors in manual analysis (Schmidt et al., 2013) and significantly reduced analysis time. These advances in turn enabled the depth and magnitude

needed for the analysis of large protein assemblies, as presented here.

Recently, a 5-subunit subcomplex of the lid (without Rpn12) was shown to bind to a chaperone-free base-CP complex in vitro (Yu et al., 2015). It is not yet clear whether this species points to an alternative route to RP formation, as it has not been reported to form naturally in vivo and it is unknown if it is competent for assembly into full 26S proteasomes. However, alternative routes to RP formation are not ruled out by our data. For example, the full lid bound to a subset of base subunits was isolated from bovine erythrocytes and shown to be capable of assembly into full RPs (Thompson et al., 2009). Alternatively, RPs or 26S proteasomes might be partially disassembled under some conditions (Fane and Prevelige, 2003; Nanduri et al., 2015).

As engagement of the LP2 helical bundle by the Rpn12 C-terminal helix was sufficient to promote interaction between LP2 and base independent of the rest of Rpn12, an allosteric signal is likely transmitted through the helical bundle. Rpn12 binding may reposition this bundle in a manner conducive for base binding. Further structural studies will be necessary to identify the specific subunit reconfigurations that drive lid-base joining. Another important challenge will be to determine the allosteric path of signal transduction from the Rpn12 C-terminal helix to the rest of the lid during conformational switching.

The Rpn12-Induced Conformational Rearrangement Is Reminiscent of Viral Maturation

Several parallels can be drawn between proteasome biogenesis and the assembly of viruses. As with the proteasomal CP and RP base, assembly of viral capsids frequently depends on exogenous chaperone proteins to form functional structures with the proper subunit arrangements (Fane and Prevelige, 2003). Further, in analogy to lid assembly and lid-base joining, incorporation of major viral subcomplexes also follows hierarchical assembly orders. For example, the head, base, and tail fibers of the T4 bacteriophage each assemble independently prior to joining together (Aksyuk and Rossmann, 2011). Finally, the capsids of many class I viruses undergo dramatic conformational rearrangements upon completion of distinct assembly events (Mateu, 2013). These rearrangements are coupled to the assembly state either via proteolytic cleavage reactions or by allosteric contacts analogous to those observed for LP2 and Rpn12. They in turn license the capsid for subsequent assembly steps, such as viral DNA packaging or attachment to the tail. Thus, while the biochemical mechanisms differ, these functional themes are shared between highly disparate macromolecular complexes and likely contribute to the faithful biogenesis of other multisubunit assemblies.

Our multipronged approach to identifying structural differences between assembly intermediates should prove valuable in future efforts to understand conformational switches and their role in proteasome assembly. The ability to reconstitute the RP in vitro from fully recombinant subunits will greatly facilitate biochemical and biophysical analyses of these processes. This approach should be readily extendable to studies of the assembly and action of other large multisubunit complexes.

EXPERIMENTAL PROCEDURES

Plasmids

Plasmids used in this study are listed in Table S2. Generation of multigene operons for bacterial expression was done as described (Tomko and Hochstrasser, 2014). All mutations were first introduced into host plasmids containing individual subunit expression cassettes, confirmed by DNA sequencing, and then transferred by subcloning into the appropriate operons.

Recombinant Protein Expression and Purification

All purified proteins were derived from the *S. cerevisiae* coding sequences and were purified either from *S. cerevisiae* or from *E. coli* as indicated in the text. All proteins and complexes were affinity purified using conventional techniques, followed by gel filtration. See also Supplemental Experimental Procedures.

Fluorescence Anisotropy Measurements

A peptide with residues 254–272 of Rpn12 followed by a 5,6-carboxyfluorescein-labeled lysine (NH_2-DQKTNIIEKAMDYAISIENIK*-CO_2H, herein referred to as N12pep) was synthesized by Elim Biopharmaceuticals. Fluorescent peptide (1 μM) was incubated in Lid Buffer alone, with 1 μM LP2, 5 μM Rpn12, or both at 25°C for 20 min. Fluorescence polarization was then measured on a two-channel fluorometer (PTI) with linear polarizers with excitation at 485 nm. The change in anisotropy compared to peptide alone was calculated using the average of the anisotropy values over the emission range 513–528 nm.

Assembly Assays

LP2 and its derivatives, lid, recombinant base precursor, and recombinant Rpn12 were diluted together to 1 μM each along with 10 μM Rpn10 in 26S buffer (50 mM Tris•HCl [pH 7.5], 5 mM $MgCl_2$, 10% glycerol, 1 mM ATP) unless otherwise noted. After incubation at 30°C for 20 min, aliquots of each reaction were separated by native polyacrylamide gel electrophoresis (native PAGE) at 100 V at 4°C as described previously (Tomko and Hochstrasser, 2014). Native PAGE-separated proteins were transferred to PVDF membranes and subjected to immunoblot analysis.

Negative-Stain Electron Microscopy

LP2 and Lid complexes were diluted to ~50 nM in Buffer A (50 mM Tris•HCl [pH 7.5], 150 mM NaCl, 5 mM $MgCl_2$, 10% glycerol) immediately prior to applying the sample to glow-discharged 400 mesh continuous carbon grids. After staining with 2% (w/v) uranyl acetate, the residual stain was blotted off, and the samples were air-dried in a fume hood. Data were acquired using a Tecnai F20 Twin transmission electron microscope at 120 keV at a nominal magnification of 80,000× (1.45 Å at the specimen level) using low-dose exposures (~20 e^- Å^{-2}) with a randomly set defocus ranging from −0.5 to −1.3 μm. A total of 150–600 images of each LP2/lid complex sample were automatically recorded on a Gatan 4k × 4k CCD camera using the MSI-Raster application within the automated macromolecular microscopy software LEGINON (Suloway et al., 2005). Specifically, 600, 510, 150, 150, and 300 micrographs were acquired of LP2, Lid, LP2 supplemented with purified Rpn12, LP2 with EGFP-Rpn6 complexes, and LP2 with peptide, respectively. See also Supplemental Experimental Procedures.

Cross-linking and Sample Preparation for Quantitative Cross-linking/Mass Spectrometry

LP2 or lid (100 μg) purified from yeast in Buffer HA (50 mM HEPES•NaOH, 150 mM NaCl, 5 mM $MgCl_2$, 10% glycerol) were each cross-linked with bis[sulfosuccinimidyl] suberate-d0 (BS^3-d0) and its deuterated form bis[sulfosuccinimidyl] 2,2,7,7-suberate-d4 (BS^3-d4) (Thermo Fisher) at 1:200 protein to cross-linker. The reaction was incubated at 30°C for 1 hr and quenched with 2 M glycine (pH 7.0) for 30 min at room temperature. Monomeric cross-linked lid and LP2 were isolated via SDS-PAGE and in-gel digested using trypsin (Chen et al., 2010). After digestion, cross-linked lid and LP2 samples were mixed in 1:1 molar ratio, yielding two samples for quantitative analysis: lid+BS^3-d0/LP2+BS^3-d4 and lid+BS^3-d4/LP2+BS^3-d0. Peptides were fractionated by strong cation exchange chromatography and

desalted using C18-StageTips for LC-MS/MS (Chen et al., 2010). See also Supplemental Experimental Procedures.

ACCESSION NUMBERS

The accession number for the LP2 EM density map reported in this paper is EMD: 3136.

SUPPLEMENTAL INFORMATION

Supplemental Information includes Supplemental Experimental Procedures, four figures, two tables, and one data file and can be found with this article online at http://dx.doi.org/10.1016/j.cell.2015.09.022.

AUTHOR CONTRIBUTIONS

R.J.T. Jr. and M.H. conceived all experiments and wrote the manuscript with feedback from all authors. R.J.T. Jr. performed all protein purifications, cross-linking, assembly assays, and fluorescence anisotropy experiments. Z.A.C. and J.R. performed all mass spectrometry and associated data analysis. D.W.T. performed all EM analyses with advice from H.-W.W.

ACKNOWLEDGMENTS

We thank Rui Zhang for helpful comments, Eva Nogales for use of her electron microscopy resources, Andreas Martin for base expression plasmids, and Dan Finley for antibodies. This work was supported by NIH grant GM083050 to M.H and the Wellcome Trust (Senior Research Fellowship 103139 and Centre core grant 092076) to J.R. R.J.T. Jr. was supported in part by an American Cancer Society postdoctoral fellowship. D.W.T. is a Damon Runyon Fellow supported by the Damon Runyon Cancer Research Foundation (DRG- 2218-15).

Received: April 3, 2015
Revised: June 19, 2015
Accepted: August 19, 2015
Published: October 8, 2015

REFERENCES

Aksyuk, A.A., and Rossmann, M.G. (2011). Bacteriophage assembly. Viruses *3*, 172–203.

Beckwith, R., Estrin, E., Worden, E.J., and Martin, A. (2013). Reconstitution of the 26S proteasome reveals functional asymmetries in its AAA+ unfoldase. Nat. Struct. Mol. Biol. *20*, 1164–1172.

Chen, Z.A., Jawhari, A., Fischer, L., Buchen, C., Tahir, S., Kamenski, T., Rasmussen, M., Lariviere, L., Bukowski-Wills, J.C., Nilges, M., et al. (2010). Architecture of the RNA polymerase II-TFIIF complex revealed by cross-linking and mass spectrometry. EMBO J. *29*, 717–726.

Cheriyath, V., Jacobs, B.S., and Hussein, M.A. (2007). Proteasome inhibitors in the clinical setting: benefits and strategies to overcome multiple myeloma resistance to proteasome inhibitors. Drugs R D. *8*, 1–12.

Cox, J., and Mann, M. (2008). MaxQuant enables high peptide identification rates, individualized p.p.b.-range mass accuracies and proteome-wide protein quantification. Nat. Biotechnol. *26*, 1367–1372.

Crawford, L.J., and Irvine, A.E. (2013). Targeting the ubiquitin proteasome system in haematological malignancies. Blood Rev. *27*, 297–304.

Dou, Q.P., and Zonder, J.A. (2014). Overview of proteasome inhibitor-based anti-cancer therapies: perspective on bortezomib and second generation proteasome inhibitors versus future generation inhibitors of ubiquitin-proteasome system. Curr. Cancer Drug Targets *14*, 517–536.

Estrin, E., Lopez-Blanco, J.R., Chacón, P., and Martin, A. (2013). Formation of an intricate helical bundle dictates the assembly of the 26S proteasome lid. Structure *21*, 1624–1635.

Fane, B.A., and Prevelige, P.E., Jr. (2003). Mechanism of scaffolding-assisted viral assembly. Adv. Protein Chem. *64*, 259–299.

Finley, D. (2009). Recognition and processing of ubiquitin-protein conjugates by the proteasome. Annu. Rev. Biochem. *78*, 477–513.

Fischer, L., Chen, Z.A., and Rappsilber, J. (2013). Quantitative cross-linking/mass spectrometry using isotope-labelled cross-linkers. J. Proteomics *88*, 120–128.

Fukunaga, K., Kudo, T., Toh-e, A., Tanaka, K., and Saeki, Y. (2010). Dissection of the assembly pathway of the proteasome lid in Saccharomyces cerevisiae. Biochem. Biophys. Res. Commun. *396*, 1048–1053.

Glickman, M.H., Rubin, D.M., Coux, O., Wefes, I., Pfeifer, G., Cjeka, Z., Baumeister, W., Fried, V.A., and Finley, D. (1998). A subcomplex of the proteasome regulatory particle required for ubiquitin-conjugate degradation and related to the COP9-signalosome and eIF3. Cell *94*, 615–623.

Hofmann, L., Saunier, R., Cossard, R., Esposito, M., Rinaldi, T., and Delahodde, A. (2009). A nonproteolytic proteasome activity controls organelle fission in yeast. J. Cell Sci. *122*, 3673–3683.

Kock, M., Nunes, M.M., Hemann, M., Kube, S., Dohmen, R.J., Herzog, F., Ramos, P.C., and Wendler, P. (2015). Proteasome assembly from 15S precursors involves major conformational changes and recycling of the Pba1-Pba2 chaperone. Nat. Commun. *6*, 6123.

Lander, G.C., Estrin, E., Matyskiela, M.E., Bashore, C., Nogales, E., and Martin, A. (2012). Complete subunit architecture of the proteasome regulatory particle. Nature *482*, 186–191.

Lingaraju, G.M., Bunker, R.D., Cavadini, S., Hess, D., Hassiepen, U., Renatus, M., Fischer, E.S., and Thomä, N.H. (2014). Crystal structure of the human COP9 signalosome. Nature *512*, 161–165.

Liu, C.W., Li, X., Thompson, D., Wooding, K., Chang, T.L., Tang, Z., Yu, H., Thomas, P.J., and DeMartino, G.N. (2006). ATP binding and ATP hydrolysis play distinct roles in the function of 26S proteasome. Mol. Cell *24*, 39–50.

Mateu, M.G. (2013). Assembly, stability and dynamics of virus capsids. Arch. Biochem. Biophys. *531*, 65–79.

Matyskiela, M.E., Lander, G.C., and Martin, A. (2013). Conformational switching of the 26S proteasome enables substrate degradation. Nat. Struct. Mol. Biol. *20*, 781–788.

Nanduri, P., Hao, R., Fitzpatrick, T., and Yao, T.P. (2015). Chaperone-mediated 26S proteasome remodeling facilitates free K63 ubiquitin chain production and aggresome clearance. J. Biol. Chem. *290*, 9455–9464.

Nyquist, K., and Martin, A. (2014). Marching to the beat of the ring: polypeptide translocation by AAA+ proteases. Trends Biochem. Sci. *39*, 53–60.

Pathare, G.R., Nagy, I., Bohn, S., Unverdorben, P., Hubert, A., Körner, R., Nickell, S., Lasker, K., Sali, A., Tamura, T., et al. (2012). The proteasomal subunit Rpn6 is a molecular clamp holding the core and regulatory subcomplexes together. Proc. Natl. Acad. Sci. USA *109*, 149–154.

Ravid, T., and Hochstrasser, M. (2008). Diversity of degradation signals in the ubiquitin-proteasome system. Nat. Rev. Mol. Cell Biol. *9*, 679–690.

Richardson, P.G., Barlogie, B., Berenson, J., Singhal, S., Jagannath, S., Irwin, D., Rajkumar, S.V., Srkalovic, G., Alsina, M., Alexanian, R., et al. (2003). A phase 2 study of bortezomib in relapsed, refractory myeloma. N. Engl. J. Med. *348*, 2609–2617.

Rinaldi, T., Pick, E., Gambadoro, A., Zilli, S., Maytal-Kivity, V., Frontali, L., and Glickman, M.H. (2004). Participation of the proteasomal lid subunit Rpn11 in mitochondrial morphology and function is mapped to a distinct C-terminal domain. Biochem. J. *381*, 275–285.

Rinaldi, T., Hofmann, L., Gambadoro, A., Cossard, R., Livnat-Levanon, N., Glickman, M.H., Frontali, L., and Delahodde, A. (2008). Dissection of the carboxyl-terminal domain of the proteasomal subunit Rpn11 in maintenance of mitochondrial structure and function. Mol. Biol. Cell *19*, 1022–1031.

Schmidt, C., Zhou, M., Marriott, H., Morgner, N., Politis, A., and Robinson, C.V. (2013). Comparative cross-linking and mass spectrometry of an intact F-type ATPase suggest a role for phosphorylation. Nat. Commun. *4*, 1985.

Sharon, M., Taverner, T., Ambroggio, X.I., Deshaies, R.J., and Robinson, C.V. (2006). Structural organization of the 19S proteasome lid: insights from MS of intact complexes. PLoS Biol. *4*, e267.

Sharon, M., Witt, S., Glasmacher, E., Baumeister, W., and Robinson, C.V. (2007). Mass spectrometry reveals the missing links in the assembly pathway of the bacterial 20 S proteasome. J. Biol. Chem. *282*, 18448–18457.

Suloway, C., Pulokas, J., Fellmann, D., Cheng, A., Guerra, F., Quispe, J., Stagg, S., Potter, C.S., and Carragher, B. (2005). Automated molecular microscopy: the new Leginon system. J. Struct. Biol. *151*, 41–60.

Thompson, D., Hakala, K., and DeMartino, G.N. (2009). Subcomplexes of PA700, the 19 S regulator of the 26 S proteasome, reveal relative roles of AAA subunits in 26 S proteasome assembly and activation and ATPase activity. J. Biol. Chem. *284*, 24891–24903.

Tomko, R.J., Jr., and Hochstrasser, M. (2011). Incorporation of the Rpn12 subunit couples completion of proteasome regulatory particle lid assembly to lid-base joining. Mol. Cell *44*, 907–917.

Tomko, R.J., Jr., and Hochstrasser, M. (2013). Molecular architecture and assembly of the eukaryotic proteasome. Annu. Rev. Biochem. *82*, 415–445.

Tomko, R.J., Jr., and Hochstrasser, M. (2014). The intrinsically disordered Sem1 protein functions as a molecular tether during proteasome lid biogenesis. Mol. Cell *53*, 433–443.

Unverdorben, P., Beck, F., Śledź, P., Schweitzer, A., Pfeifer, G., Plitzko, J.M., Baumeister, W., and Förster, F. (2014). Deep classification of a large cryo-EM dataset defines the conformational landscape of the 26S proteasome. Proc. Natl. Acad. Sci. USA *111*, 5544–5549.

Verma, R., Aravind, L., Oania, R., McDonald, W.H., Yates, J.R., 3rd, Koonin, E.V., and Deshaies, R.J. (2002). Role of Rpn11 metalloprotease in deubiquitination and degradation by the 26S proteasome. Science *298*, 611–615.

Witt, S., Kwon, Y.D., Sharon, M., Felderer, K., Beuttler, M., Robinson, C.V., Baumeister, W., and Jap, B.K. (2006). Proteasome assembly triggers a switch required for active-site maturation. Structure *14*, 1179–1188.

Worden, E.J., Padovani, C., and Martin, A. (2014). Structure of the Rpn11-Rpn8 dimer reveals mechanisms of substrate deubiquitination during proteasomal degradation. Nat. Struct. Mol. Biol. *21*, 220–227.

Yu, Z., Livnat-Levanon, N., Kleifeld, O., Mansour, W., Nakasone, M.A., Castaneda, C.A., Dixon, E.K., Fushman, D., Reis, N., Pick, E., and Glickman, M.H. (2015). Base-CP proteasome can serve as a platform for stepwise lid formation. Biosci. Rep. *35*, e00194.

Supplemental Figures

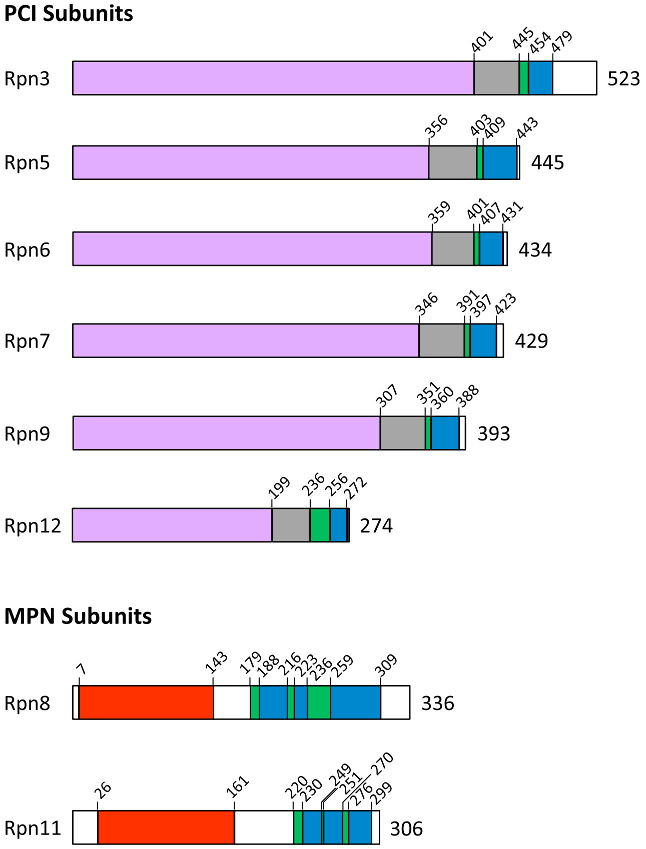

Figure S1. Domain Architecture of Proteasome Lid Subunits, Related to Figure 1

The domain structures of PCI and MPN lid subunits are shown. The domains of the six PCI subunits are colored as follows: pink, N-terminal domain; gray, winged helix domain; green, linker region; blue, C-terminal helix contributing to the lid helical bundle. The domains of the MPN domain-containing subunits, Rpn8 and Rpn11, are colored as the PCI subunits, except the MPN domain is shown in red. The number following each bar diagram indicates the total number of amino acids in the subunit, and the numbers at the domain boundaries mark the predicted first amino acid in each domain. Domain boundaries were inferred based on integrated analyses of the subunits' predicted secondary structures using PSIPred, comparisons to protein domain databases such as SMART (Simple Modular Architecture Research Tool), and the modeled tertiary structures in the cryo-EM structure of the proteasome (PDB 4CR2).

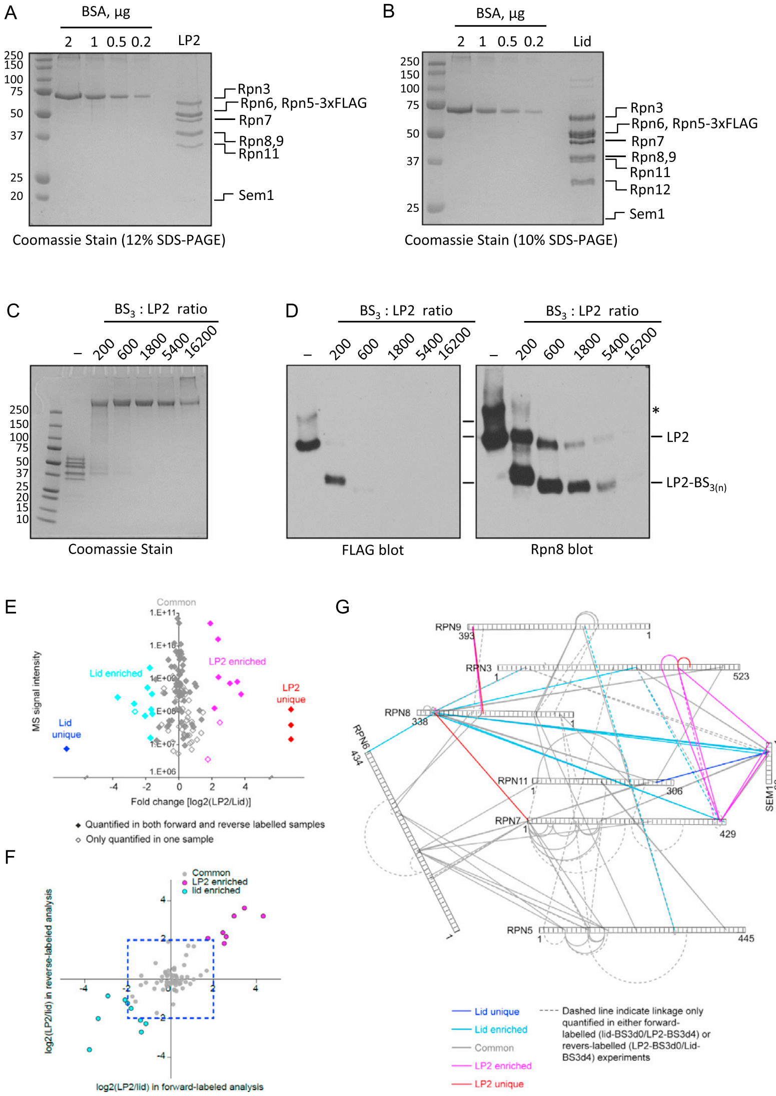

Figure S2. Validation of Quantitative Cross-linking-Mass Spectrometry Strategy, Related to Figure 2

(A and B) Purifications of LP2 (A) and lid (B) from yeast are shown.

(C and D) Titration of BS_3 cross-linker. LP2 was treated with increasing molar ratios of BS_3 to LP2 for 1 hr at 30°C, followed by quenching of unreacted BS_3 with 2 M glycine, pH 7.4. Proteins were then analyzed by SDS-PAGE (C) or native PAGE-immunoblotting (D). Essentially identical results were obtained for lid (not shown). In (D), loss of reactivity to the anti-FLAG antibody likely results from modification of lysine(s) in the epitope DYKDDDDK by BS_3. A similar effect is observed in the Rpn8 blot at the highest ratios of cross-linker to LP2. The increased mobility of LP2 in the native gel upon reaction with BS_3 is likely due to the loss of positively charged lysine side chains that would retard the protein complex in the electric field, as well as a potentially more compact conformation enforced by cross-linking. Note that the previously observed native PAGE-induced dimer of LP2 (asterisk) is not enriched with the cross-linker molar ratios used herein, suggesting that the vast majority of LP2 cross-links are intramolecular.

(E) Fold changes [Log2(LP2/lid)] of 122 quantified cross-links were plotted against their signal intensities. In total, 81 cross-links were quantified in both forward and reverse-labeled experiment (solid dots) whereas 41 cross-links were only quantified in one of two experiments (hollow dots). These quantified cross-links have been divided into 5 subgroups and colored accordingly: four are unique to the LP2 sample (red); one is unique to the lid sample (blue); among 117 cross-links that were observed in both LP2 and lid, 98 showed no significant difference on yields in both conformations (gray), whereas nine cross-links were significantly upregulated in the LP2 sample ($p < 0.05$, magenta), and ten were significantly upregulated in the lid sample ($p < 0.05$, cyan).

(F) Reproducibility of QCLMS analysis. For 77 cross-links that have been quantified in both forward and reverse-labeled experiments (not including LP2-unique and lid-unique cross-links), their fold change ([log2(LP2/lid)] in the forward-labeled experiment are plotted against their counterpart in the reverse-labeled experiment. Cross-links are colored according to their quantitation subgroups as defined in (E).

(G) Cross-linking network of lid/LP2 subunits. Proteins are shown in bars. Cross-links that were quantified in both forward and reverse-labeled experiments are shown as solid lines; while cross-links that were only quantified in one of two experiments are shown as a dashed line. All cross-links are colored according to their quantitation subgroups as defined in (E).

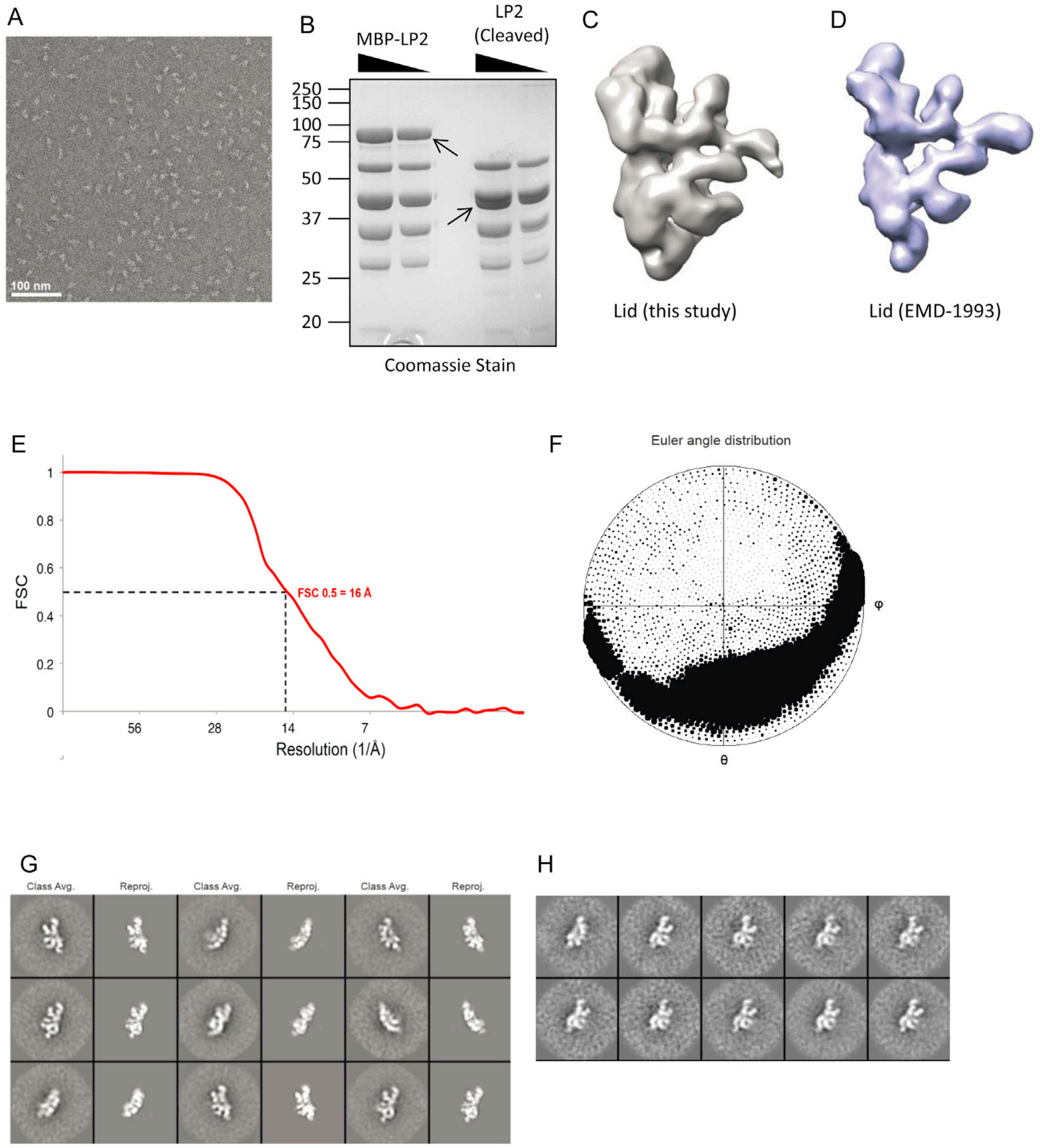

Figure S3. Supporting Data for EM Analysis of LP2, Related to Figure 4

(A) Representative micrograph of negatively stained LP2. The scale bar indicates 100 nm.

(B) SDS-PAGE analysis of MBP-tagged LP2 before and after removal of the MBP tag. Arrows indicate the position of MBP-Rpn6 or untagged Rpn6.

(C and D) Extension of the Rpn6 N terminus was observed both in 3D reconstructions of lid performed in this study (C) and from EMD-1993 (D), indicating that the presence or absence of Rpn12 in the complex, rather than sample preparation, accounts for the lack of density for the Rpn6 N terminus in LP2 reconstructions.

(E) Fourier shell correlation (FSC) curve for the final LP2 reconstruction, showing the resolution to be ~16 Å using the 0.5 FSC criterion.

(F) Euler angle distribution for the final reconstruction.

(G) Reference-free 2D class averages of LP2 (first, third, fifth columns) matched to reprojections of the final reconstruction (second, fourth, and sixth columns). The width of the boxes is ~430 Å.

(H) Reference-free 2D class averages of purified recombinant LP2.

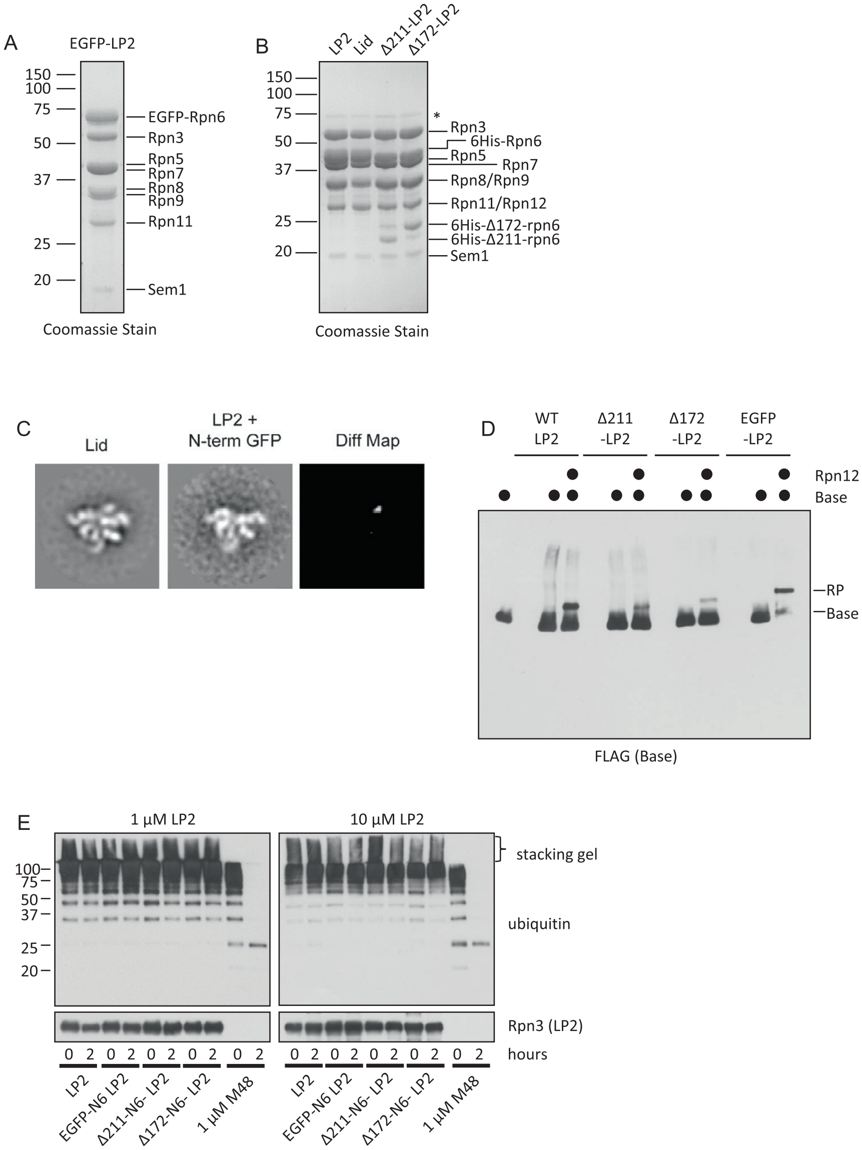

Figure S4. Biochemical Analysis of LP2 Mutants, Related to Figure 6 and Discussion

(A) SDS-PAGE analysis of LP2 containing N-terminally EGFP-tagged Rpn6.

(B) SDS-PAGE analysis of LP2, LP2 containing Rpn6 N-terminal truncation mutants, and lid. Asterisk, a contaminating *E. coli* protein.

(C) From left to right: representative reference-free 2D class average of lid, the corresponding reference-free 2D class average of LP2 with the fusion of EGFP to the N terminus of Rpn6, and a 2D difference map between the two class averages. This panel shows that the extended arm is most likely Rpn6's N terminus rather than EGFP. The width of the boxes is ~430 Å.

(D) LP2 complexes containing mutant forms of Rpn6 are proficient for RP formation. The indicated forms of LP2 were incubated with 1 mM ATP, 1 μM recombinant Base precursor and 10 μM Rpn10, with or without 1 μM recombinant Rpn12 for 20 min at 30°C followed by native PAGE and immunoblotting against FLAG-tagged Rpt1.

(E) LP2 and LP2 mutants do not show appreciable deubiquitinating activity toward polyubiquitinated Sic1. The indicated forms of LP2 at concentrations of 1 μM or 10 μM (left and right panels, respectively) were incubated with polyubiquitinated Sic1 (Saeki et al., 2005) for zero or two hours before analysis by SDS-PAGE and immunoblotting against ubiquitin or Rpn3. As a control for deubiquitination, polyubiquitinated Sic1 was also incubated with 1 μM of the murine cytomegalovirus M48 deubiquitinase (Schlieker et al., 2007). The high activity of this deubiquitinase resulted in a loss of some polyubiquitinated Sic1 (evident in the stacking gel) in the seconds between addition of the enzyme and quenching of the reaction with Laemmli buffer and boiling.

Cell
Supplemental Information

A Single α Helix Drives Extensive Remodeling of the Proteasome Lid and Completion of Regulatory Particle Assembly

Robert J. Tomko, Jr., David W. Taylor, Zhuo A. Chen, Hong-Wei Wang, Juri Rappsilber, and Mark Hochstrasser

Supplemental Experimental Procedures

Purified LP2 and lid from yeast were prepared exactly as described previously (Tomko and Hochstrasser, 2011). Recombinant Rpn10 was purified on Ni-NTA resin (Qiagen) via an N-terminal 6His tag, followed by Sephacryl S-200 chromatography in Lid Buffer (50 mM HEPES•NaOH, pH 7.5, 100 mM NaCl, 100 mM KCl, 5% glycerol, 1 mM DTT). Recombinant base precursor bearing an N-terminal FLAG tag on Rpt1 and an N-terminal 6His tag on Rpt3 was expressed and purified exactly as described previously (Beckwith et al., 2013). Base precursor bearing an N-terminal MBP tag on Rpt1 and a C-terminal 6His tag on Rpn2 was purified exactly as for FLAG-tagged base precursor, except that the eluates from the Ni^{2+} affinity step were bound to amylose resin (NEB Corp.) in Base Buffer (50 mM HEPES•NaOH, pH 7.5, 100 mM NaCl, 100 mM KCl, 10 mM $MgCl_2$, 0.5 mM EDTA, 10% glycerol, 1 mM DTT), washed with Base Buffer, and eluted with Base Buffer containing 10 mM maltose instead of FLAG peptide.

Recombinant LP2, lid, and their derivatives were expressed in BL21-STAR (DE3) cells (Invitrogen) in Terrific Broth overnight at 16°C. For purification of complexes harboring an N-terminal MBP tag on Rpn6, cells in Lid Buffer supplemented with protease inhibitors were lysed using an M-110EH microfluidizer (Microfluidics Corp.). Lysates were cleared by centrifugation for 20 min at 30,000 x *g* at 4°C and bound to amylose resin. After washing extensively with Lid Buffer, proteins were eluted with 10 mM maltose in Lid Buffer, and separated by Superose 6 chromatography (GE Life Sciences) isocratically in Lid Buffer. The MBP tag was removed with human rhinovirus 3C protease overnight at 4°C, and the protease and cleaved tag were removed by Superose 6 chromatography as above. Polyhistidine-tagged proteins were purified as above, except that lysis and wash buffers for affinity purification contained 20 mM imidazole, Ni-NTA

resin was substituted for amylose resin, and proteins were eluted with Lid Buffer containing 500 mM imidazole. Complexes were then further purified by Superose 6 chromatography in Lid Buffer. All proteins were concentrated via centrifugal filtration using concentrators (Amicon) with an appropriate molecular weight cut-off for retention of the desired species, flash-frozen in liquid nitrogen, and stored at -80°C until use.

Single-particle pre-processing, alignment, and classification. All image pre-processing and two-dimensional classification was performed in Appion (Lander et al., 2009) as described previously (Lander et al., 2012). The contrast transfer function (CTF) of each micrograph was estimated, and particles were selected concurrently with data collection using ACE2 (Mallick et al., 2005) and a template-based particle picker (Roseman, 2004), respectively. Reference-free 2D class averages of lid and reference-free 2D class averages of LP2 were used to minimize reference bias. Micrograph phases were corrected using ACE2 (Mallick et al., 2005), and the negatively stained LP2/lid particles were extracted using a 288 × 288-pixel box size. The particle stacks were binned by a factor of 2 for processing, and particles were normalized to remove pixels whose values were above or below 4.5-σ of the mean pixel value using XMIPP (Sorzano et al., 2004). Particle stacks were binned again by a factor of 2 and subjected to five rounds of iterative multivariate statistical analysis (MSA) and multi-reference alignment (MRA) using the IMAGIC (van Heel et al.) software package to generate reference-fee class averages. The resulting set of class averages for each species was normalized using ‘proc2d’ in EMAN (Ludtke et al., 1999). The EMAN classification program ‘classesbymra’ was used to match each LP2 complex to the best-matching lid class average based on cross-correlation coefficients.

3D reconstruction and analysis. All three-dimensional reconstructions were performed using an iterative projection-matching refinement with libraries from the EMAN2 and SPARX software packages (Hohn et al.; Tang et al.). Refinement of the initial models began using an angular increment of 25°, progressing down to 4° for all reconstructions. The initial model used for each dataset was EMDB-1993 (Lander et al., 2012) low-pass filtered to 60-Å resolution. In an alternative approach for the lid, we used a low-pass filtered model of the other structure (LP2) after refinement as an initial model for the above-mentioned projection matching refinement. This led to an EM density with similar structural features (data not shown). Estimated resolutions were determined by calculating the Fourier shell correlation (FSC) between each of the back-projected volumes of two equally sized data sets. The final reconstructions of LP2 and lid showed structural features to ~16 Å and ~22 Å, respectively, based on the 0.5 Fourier shell correlation criterion (Fig. S3E). Reprojections of the final three-dimensional reconstruction of LP2 showed excellent agreement with the reference-free class averages (Fig. S3G) and displayed a large distribution of Euler angles, despite some preferential orientations of the particles on the carbon film (Fig. S3F).

The final reconstruction was segmented using Segger (Pintilie et al., 2010) in Chimera (Pettersen et al., 2004) based on inspection of the similarities between our LP2 reconstruction and the previously determined structure and segmentation of the lid (Lander et al., 2012). LP2 was superimposed onto the pseudo-atomic model of the 26S proteasome using a multi-step procedure. First, the previously determined lid structure was segmented and then docked into the lid subunits on the pseudo-atomic model of the 26S proteasome. Then, we docked our LP2 reconstruction into the lid density by optimizing the cross-correlation coefficient between the two EM densities. The N-terminal domain of Rpn6 from the pseudo-atomic model is not

accommodated by either of the EM structures, likely due to structural changes that occur upon Lid incorporation into the core particle as described previously (Lander et al., 2012).

Mass spectrometric analysis. We first excised cross-linked products of lid and LP2 from SDS-PAGE gels to obtain monomers, and not higher-order cross-linked forms, of each complex. The monomers of cross-linked lid and LP2 were then mixed after tryptic digestion. A precise 1:1 mixing was achieved based on a preliminary experiment in which we mixed small aliquots of each digested sample, conducted mass spectrometric measurements to determine actual molar ratios, and then used this information to normalize the protein amounts for mixing in the crosslink mapping analysis. Specifically, the digested samples were mixed in an approximately 1:1 ratio as estimated by the approximate protein concentrations used in the experiment. A short LC-MS analysis was conducted without any special instrument settings in order to detect linear modified peptides. Peptides were identified using Mascot version 2.4 and allowing for the cross-linker as a variable modification. Cross-linker modified peptides were then quantified by help of XiQ using the parameters described previously (Fischer et al., 2013). The median ratio for the modified peptides was used to correct the initial mixing ratio. Figure 2B illustrates the success of this procedure, as the quantified linkages center around log 2 = 0, i.e. 1:1 mixing ratio. To evaluate digestion efficiency, we used the number of uncut trypsin-cleavage sites in identified linear (non-cross-linked peptides) as an indicator of incomplete digestion. Few missed cleavages were found, suggesting similarly high and efficient digestion for both lid and LP2 samples.

LC-MS/MS analysis was conducted using a hybrid quadrupole-Orbitrap mass spectrometer (Q Exactive, Thermo Fisher Scientific). Peptides were separated on a reversed-phase analytical column (Chen et al., 2010). Mobile phase A consisted of water and 0.1% v/v

formic acid. Mobile phase B consisted of 80% v/v acetonitrile and 0.1% v/v formic acid. Peptides were loaded at a flow rate of 500 nl/min and eluted at 200 nl/min. The separation gradient consisted of a linear increase from 2% mobile phase B to 40% mobile phase B in 79 minutes and a subsequent linear increase to 95% B over 11 minutes. Eluted peptides were directly sprayed into the Q Exactive mass spectrometer. MS data were acquired in the data-dependent mode. For each acquisition cycle, the MS spectrum was recorded in the Orbitrap at 70,000 resolution. The ten most intense ions in the MS spectrum, with a precursor charge state 3+ or greater, were fragmented by Higher Energy Collision Induced Dissociation (HCD). The fragmentation spectra were thus recorded in the Orbitrap at 35,000 resolution. Dynamic exclusion was enabled, with single-repeat count and a 40 second exclusion duration.

Identification of cross-linked peptides. The raw mass spectrometric data files were processed into peak lists using MaxQuant version 1.2.2.5 (Cox and Mann, 2008) with default parameters, except that “Top MS/MS Peaks per 100 Da” was set to 20. The peak lists were searched against sequences of all lid subunits using Xi software (ERI, Edinburgh) for identification of cross-linked peptides. Search parameters were as follows: MS accuracy, 6 ppm; MS2 accuracy, 20 ppm; enzyme, trypsin; specificity, fully tryptic; allowed number of missed cleavages, four; cross-linker, BS^3/BS^3-d4; fixed modifications, carbamidomethylation on cysteine; variable modifications, oxidation on methionine. The linkage specificity for BS^3 was assumed to be for lysine, serine, threonine, tyrosine and protein N-termini. All identified cross-linked peptides were validated manually based on their MS2 spectra and MS signals (either as doublet signals or singlet signals with corresponding mass shift in label-swapped replica analysis Fig 2A).

Quantitation of cross-linking data. Identified cross-linked peptides were quantified based on their MS signals. Quantitative proteomics software tool Pinpoint was used to retrieve intensities of both light and heavy signals for each cross-linked peptides in an automated manner. An input library for cross-linked peptides was constructed according to "General Spectral Library Format for Pinpoint Comma Separated Values" (Thermo Fisher Scientific). The three most abundant isotope peaks were used for quantitation. The error tolerance for precursor m/z was set to 6ppm. Signals were only accepted within a window of retention time (defied in spectral library) ±10 minutes. Manual inspection was carried out to ensure the correct isolation of elution peaks. For each cross-linked peptide, elution peak areas of the light and the heavy signals were measured and signal fold changes were calculated as log2 (LP2/lid). Quantitation data was subsequently summarized at the level of unique residue pairs (cross-links). A cross-link was defined as a unique cross-link in either lid or LP2 only if all its supporting cross-linked peptides were observed as according singlet signals. Otherwise, cross-links were regarded as being observed in both particles. For a cross-link, the signal fold change was defined as the median of all its supporting cross-linked peptides. Only those cross-links that were consistently quantified in both paired replicas (with label-swap) were accepted for subsequent structural analysis. Singlet cross-links were further confirmed by a mass shift of 4 Da, resulting from the label-swap. For cross-links observed as a doublet, the average of fold ratios from replica analyses was reported. All quantified cross-links were listed in Supplementary Table S1. Cross-links that were significantly enriched in lid or LP2 were determined using "Significance A" test from the standard proteomics data analysis tool Perseus (version 1.4.1.2) (Cox and Mann, 2008) based on log2(LP2/lid) values. The following parameters were used for the test: "Side": both; "Use for truncation": P value; "Threshold value": 0.05.

Mapping and comparing cross-linking data with the pseudoatomic model of lid

To compare cross-linking data on the isolated lid with the structure of the lid in the context of the 26S proteasome, we mapped the identified and quantified lid cross-links onto the pseudoatomic structure of lid in the 26S proteasome structure (PDB 4CR2). Using PyMol (version 1.2b5) (DeLano, 2002), cross-links were displayed as lines between the C-α atoms of linked residues (cross-links were displayed only when both linked residues were present in the structure 4CR2). The distance of a cross-linked residue pair was measured between the C-α atoms. Measured distances of linked residue pairs in the pseudoatomic structure were compared to a theoretical cross-linking limit, which was calculated as side-chain length of cross-linked residues plus the spacer length of the cross-linker (11.4 Å for BS3). An additional 2 Å was added for each residue as an allowance for residue displacement from the pseudoatomic structure. The following side chain lengths were used for the calculation: 6.0 Å for lysine, 2.4 Å for threonine, 2.4 Å for serine and 6.5 Å for tyrosine. For example, for a lysine-lysine cross-link, this limit is 6+6+11.4+2+2=27.4 Å. While one might use a larger distance cut-off, we see most of our data in other studies where crystal structures were available to adhere to this upper limit. Also, the links that we observed in this study as over-length exceeded our upper limit by up to 99 Å and thus require the assumption of large conformational changes.

Supplemental Table S2 Related to Experimental Procedures. Plasmids used in this study.

**Plasmid**	**Genotype**
pRT205	pET28a-Rpn10
pRT585[a]	pET42b-Rpn3 : Sem1 : Rpn7
pRT586	pET42b-Rpn3 : Sem1 : Rpn7 : Rpn12
pRT945[a]	pCDF42b-6His-MBP-3Cx-Rpn6 : Rpn9 : Rpn11 : Rpn5 : Rpn8
pRT1010	pCDF42b-10His-EGFP-Rpn6 : Rpn9 : Rpn11 : Rpn5 : Rpn8
pRT1030	pCDF42b-6His-Rpn6 : Rpn9 : Rpn11 : Rpn5 : Rpn8
pRT1031	pCDF42b-6His-rpn6(173-434) : Rpn9 : Rpn11 : Rpn5 : Rpn8
pRT1032	pCDF42b-6His-rpn6(212-434) : Rpn9 : Rpn11 : Rpn5 : Rpn8
pRT1078	pET42b-Rpn3 : Sem1 : Rpn7 : rpn12(E271K)
pRT1096[b]	pCOLADuet-1-FLAG-Rpt1 : Rpt2 : 6His-Rpt3 : Rpt5 : Rpt6 : Rpt4
pRT1097[b]	pETDuet-1-Rpn1 : Rpn2 : Rpn13
pRT1098[b]	pACYCDuet-1-Nas6 : Nas2 : Hsm3 : Rpn14
pRT1122	pET42b-6His-Cys-rpn12(C23S,D265A)
pRT1202	pETDuet-1-Rpn1 : Rpn2-6His : Rpn13
pRT1260	pCOLADuet-1-MBP-3Cx-Rpt1 : Rpt2 : Rpt3 : Rpt5 : Rpt6 : Rpt4

[a] Described in Mol Cell. 2014;53:433-43.
[b] Described in Nat Struct Mol Biol. 2013; 20:1164-72.

Note: Unless otherwise indicated, all plasmids were produced in this study. 3Cx indicates a human rhinovirus 3C protease cut site.

Supplemental References

Chen, Z.A., Jawhari, A., Fischer, L., Buchen, C., Tahir, S., Kamenski, T., Rasmussen, M., Lariviere, L., Bukowski-Wills, J.C., Nilges, M., *et al.* (2010). Architecture of the RNA polymerase II-TFIIF complex revealed by cross-linking and mass spectrometry. EMBO J *29*, 717-726.

Cox, J., and Mann, M. (2008). MaxQuant enables high peptide identification rates, individualized p.p.b.-range mass accuracies and proteome-wide protein quantification. Nat Biotechnol *26*, 1367-1372.

DeLano, W.L. (2002). The PyMOL Molecular Graphics System.

Fischer, L., Chen, Z.A., and Rappsilber, J. (2013). Quantitative cross-linking/mass spectrometry using isotope-labelled cross-linkers. J Proteomics *88*, 120-128.

Hohn, M., Tang, G., Goodyear, G., Baldwin, P.R., Huang, Z., Penczek, P.A., Yang, C., Glaeser, R.M., Adams, P.D., and Ludtke, S.J. (2007). SPARX, a new environment for Cryo-EM image processing. J Struct Biol *157*, 47-55.

Lander, G.C., Stagg, S.M., Voss, N.R., Cheng, A., Fellmann, D., Pulokas, J., Yoshioka, C., Irving, C., Mulder, A., Lau, P.W., *et al.* (2009). Appion: an integrated, database-driven pipeline to facilitate EM image processing. J Struct Biol *166*, 95-102.

Ludtke, S.J., Baldwin, P.R., and Chiu, W. (1999). EMAN: semiautomated software for high-resolution single-particle reconstructions. J Struct Biol *128*, 82-97.

Mallick, S.P., Carragher, B., Potter, C.S., and Kriegman, D.J. (2005). ACE: automated CTF estimation. Ultramicroscopy *104*, 8-29.

Pettersen, E.F., Goddard, T.D., Huang, C.C., Couch, G.S., Greenblatt, D.M., Meng, E.C., and Ferrin, T.E. (2004). UCSF Chimera--a visualization system for exploratory research and analysis. J Comput Chem *25*, 1605-1612.

Pintilie, G.D., Zhang, J., Goddard, T.D., Chiu, W., and Gossard, D.C. (2010). Quantitative analysis of cryo-EM density map segmentation by watershed and scale-space filtering, and fitting of structures by alignment to regions. J Struct Biol *170*, 427-438.

Roseman, A.M. (2004). FindEM--a fast, efficient program for automatic selection of particles from electron micrographs. J Struct Biol *145*, 91-99.

Saeki, Y., Isono, E., and Toh, E.A. (2005). Preparation of ubiquitinated substrates by the PY motif-insertion method for monitoring 26S proteasome activity. Methods Enzymol *399*, 215-227.

Schlieker, C., Weihofen, W.A., Frijns, E., Kattenhorn, L.M., Gaudet, R., and Ploegh, H.L. (2007). Structure of a herpesvirus-encoded cysteine protease reveals a unique class of deubiquitinating enzymes. Mol Cell *25*, 677-687.

Sorzano, C.O., Marabini, R., Velázquez-Muriel, J., Bilbao-Castro, J.R., Scheres, S.H., Carazo, J.M., and Pascual-Montano, A. (2004). XMIPP: a new generation of an open-source image processing package for electron microscopy. J Struct Biol *148*, 194-204.

Tang, G., Peng, L., Baldwin, P.R., Mann, D.S., Jiang, W., Rees, I., and Ludtke, S.J. (2007). EMAN2: an extensible image processing suite for electron microscopy. J Struct Biol *157*, 38-46.

van Heel, M., Harauz, G., Orlova, E.V., Schmidt, R., and Schatz, M. (1996). A new generation of the IMAGIC image processing system. J Struct Biol *116*, 17-24.
